# Integrated guidance and control design by active disturbance rejection method for high-velocity target interceptor with DCS thruster

**DOI:** 10.1038/s41598-024-52008-4

**Published:** 2024-01-14

**Authors:** Ali Chitsaz, Abolghasem Naghash, Farhad Fani Saberi

**Affiliations:** 1 Department of AeroSpace Engineering, Amirkabir University of Technology, Tehran, Iran; 2 Aerospace Science and Technology Institute, Amirkabir University of Technology, Tehran, Iran

**Keywords:** Aerospace engineering, Mechanical engineering

## Abstract

The present paper proposes a novel integrated guidance and control (IGC) method for engaging with high-speed targets such as ballistic projectiles. considering an extreme short period of terminal engagement due to high relative velocity between target and interceptor, it is particularly important for IGC law to show desirable performance in the presence of various uncertainties (e.g. variation in aerodynamic coefficients) and disturbances (e.g. target maneuver and drag). This article extends the ICG law for mismatched and feedback form equations based on the Active Disturbance Rejection Control (ADRC) method using the back-stepping technique and the Reduced-order Extended State Observer (RESO). The primary consideration is the application of thrusters on the center of mass as the Divert Control System (DCS), along with the daisy-chain technique for control allocation between the fins and thruster commands. Contrary to previous research, the filter and angle measurement error are modeled for the seeker as a crucial parameter to highlight the significance of the thruster. The simulation results indicate the efficiency of the developed method for near-miss or hit-to-kill engagement with tactical ballistic targets. It is shown that the thruster plays a significant role in high-altitude engagements, specifically in the presence of non-ideal seeker. Finally, using the Monte Carlo simulation, it is proved that adding inner loops to the developed technique will not remove the IGC’s advantage over the conventional approach and Non-singular Terminal Sliding Mode (NTSM) guidance law.

## Introduction

### Engagement with ballistic targets and its challenges

As a result of the development of ballistic missiles as high-speed vehicles, combating these missiles has been the main challenge for air defense systems. The priority of countermeasures against these targets is interception during the boost phase, followed by exit from the atmosphere at the mid-course phase. This is related to low velocity besides the large radar cross section in an early stage of flight. Later, despite the high velocity, there is no drag or maneuverability due to the lack of atmosphere. Therefore, the trajectory of the projectile will be completely predictable. In these two phases, countermeasures face numerous tactical and technological obstacles such as the need to be in the enemy’s area for engagement in the boost phase, or the existence of an extensive and powerful radar network even in other countries.

Consequently, most air defense systems intercept ballistic threats at the reentry phase. During this phase, the target has a high velocity, a small radar cross section, and the ability to change its trajectory. The first two characteristics reduce the interception time, while drag and maneuverability complicate the geometry of the engagement.

To strike ballistic targets, the incident must first be a near-miss or a hit-to-kill one; second, it must occur at high altitudes, where the target cannot maneuver and if one interceptor fails to engage the target, another one still has a chance. Additionally, the cluster or chemical warhead causes less damage to the environment. Loss of aerodynamic efficiency and an increase in the time constant of the interceptor’s autopilot are obstacles posed by engagements at high altitudes. The use of thrusters in defense systems has been investigated as a possible solution to the issues mentioned above. More explanations regarding the types of thrusters and their features are provided in Section “Thruster description”.

### IGC background and common methods

Generally, another obstacle is the limited time available during the final phase of engagement. The benefits of IGC include (a) eliminating the inevitable lag between traditional guidance and control loops, resulting in high-speed performance (b) considering interceptor dynamics (like aerodynamic capability) in guidance equations, resulting in desirable performance while preventing saturation during rapid changes in engagement geometry^1^.

Due to its robustness against uncertainties and disturbances, Sliding Mode Control (SMC) is one of the categories most commonly employed IGC design methods. In references^2,3^, the conventional SMC law is used to intercept a maneuvering target. These studies assume that the target acceleration is known, and if estimation or measurement errors exist, this method will not perform as desired. This technique is applied to a “bang-bang” interceptor in^4^. Using first-order SMC and linear matrix inequalities, a robust IGC law is proposed to intercept targets at the ground level^5^. A high-order SMC approach has been utilized in references^6,7^ to decrease control signal chattering, finite-time convergence and reduce the amount of required information about the target. Due to the feedback form of IGC equations, back-stepping and inverse dynamics control methods have been highly used in addition to the SMC method. Adaptive variants of these methods are also implemented by authors of^8,9^.

Moreover, numerical methods, including a State-Dependent Riccati Equation (SDRE)^10^ and $\theta -D$^11^, have been utilized for the three-dimensional design of IGC. Novel techniques, including the small-gain theorem^12^, and nonlinear Receding Horizon Pseudospectral Control (RHPC)^13^ have also been developed.

### Research motivation

The main motivation of this research is to develop a method to destroy a tactical ballistic target in the atmosphere. According to Section “Engagement with ballistic targets and its challenges”, the miss distance should be smaller than 1 m (for hit-to-kill interception) and the engagement should be at an altitude as high as possible. Achieving this interception accuracy requires to consider all uncertainties, disturbances and different types of target maneuvers. On the other hand, it is necessary for the developed method to eliminate the need for expensive and very high accuracy seekers. This problem includes complexities such as the existence of nonlinear terms in the equations, high drag acceleration of ballistic targets, target maneuver, change of aerodynamic coefficients due to the interaction of thruster outlet jet with free flow, the uncertainty of coefficients, disturbance torque caused by thruster and seeker error in short time interception.

The main issue identified in Section “IGC background and common methods” is the inability to deal with target acceleration, uncertainties, and disturbances simultaneously with desirable control performance. Those articles using back-stepping family or numerical methods, account for minute uncertainties and disturbances. Also the effect of some nonlinear terms such as the angle between the velocity vector and the line-of-sight is not seen in the model for simplification, and target without maneuver is assumed. Also the works addressing SMCs, face several problems: (a) In the initial research, they wanted complete information about the target maneuver, and then they tried to make the information less, but this need still exists (b) With increasing target acceleration (which occurs in ballistic targets due to high speed), a larger switching gain should be selected, which leads to chattering. (c) Despite encountering multiple disturbances, perform the simulations at low velocities (long interception time) to avoid chattering to obtain good performance, which is inefficient for intercepting tactical ballistic targets (d) The need for a high fin rate actuator.

Today, due to advancements in control strategies, more effective methods are used to deal with disturbances without causing SMC problems. Among these techniques is Disturbance Observer Based Control (DOBC), which employs a double-layer structure for removing disturbances and improves closed-loop performance. Due to complexities mentioned above, this structure as a controller can be helpful. It is no longer necessary to know the acceleration band of the target.

There are many methods in the DOBC family, one of them is Active Disturbance Rejection Control (ADRC). Classic ADRC is used for integral chain systems and matching conditions^14^. In contrast, IGC equations are mismatched and formulated in a feedback form. This challenge necessitates initiatives for the IGC structure’s implementation of this method. Also the advantage of this method is the controller’s consideration of all system-affecting factors, including nonlinearities, uncertainties, and external disturbances, as a lumped disturbance that must be estimated and compensated.

### IGC and DOBC

In^15,16^ ESO estimates target acceleration. Reference^17^ focused on ground target with negligible maneuver. It uses ESO combined with back-stepping method. Reference^18^ employed a Reduced-order Extended State Observer (RESO) filter and the back-stepping control, demonstrating that RESO has a wider bandwidth than ESO. The actuator rate saturation is not considered in the simulation, and it does not have the ability to engage with high-speed targets with high Zero Effort Miss (ZEM). SMC and super-twisting ESO combination was used for three-dimensional interception, considering the impact angle described in^19^. Due to the type of filter used along with the sliding mode structure, it cannot have the desired efficiency in a short time and at a high speed. To get a faster answer, Non-singular Terminal Sliding Mode control (NTSM) with ESO is used in^20^ for intercepting maneuvering target. To avoid chattering, the authors used nonlinear tracking differentiator that complicates the issue. In^21^, RESO combination with the back-stepping and sliding mode framework is also used. The important point of this research is to consider the delay of the actuator. However the desired performance at high speed without complicating the control structure is still neglected. Some other types of observers like nonlinear filter,^22^ and adaptive one^23^ have been employed to estimate disturbances.

### Our contributions

As reviewed in Section “IGC and DOBC” most of the research conducted in the field of combining DOBC and IGC has used the structure of back-stepping or SMC due to easy implementation. However what is neglected, is the simultaneous high maneuver target, high closing velocity and high altitude engagement, with multiple disturbances, all required to intercept a ballistic target. These constraints cause the equations to be changed for the use of Divert Control System (DCS) thruster and the requirement of simultaneous commanding to thruster and fins in the final phase arises. Also, the RESO filter is used to estimate the disturbances and uncertainties with a suitable bandwidth and compatible with the cascade control structure. The next issue is that all the cases investigated, use true value for line-of-sight rate without considering the seeker filter and measurement error in the guidance as an important source of error to reach the hit-to-kill interception. In this regard, the main contributions of the present study are summarized as follows:

- To overcome the challenges of engagement with a tactical ballistic target, we use an interceptor with a tail and a thruster on the center of mass using the control allocation algorithm in the IGC and DOBC structure.
- Contrary to all existing solutions, seeker dynamics and measurement errors are formulated and implemented. Due to the short homing phase, this error in the line-of-sight rate poses a significant difficulty in intercepting high-speed targets with a small radar cross-sectional area.
- A complete simulation was performed by sweeping ZEMs and different heights to demonstrate the effectiveness of this method versus the conventional method.

It has been mentioned in some references such as^22^ that the cascade structure in IGC has destroyed its advantage over the conventional method. In Section “Case 3” this claim is completely rejected and the reasoning is explained.

### Organization

The current paper is structured as follows: In Section “Problem formulation” a mathematical model of the thruster is developed for the engagement problem. Section “IGC law design with Reduced order ESO” discusses the design of the controller with an observer. Section “Stability analysis of the closed-loop system” demonstrates the stability of the implemented method, while Section “Seeker filter design” defines seeker dynamics and measurement error. Furthermore, Section “Simulation results” presents the results of detailed comparisons between simulations to illustrate the crucial role of the proposed method. Section  “Concluding Remarks” provides conclusion remarks, discussing the effectiveness and difficulties of proposed method for intercepting ballistic targets.

## Problem formulation

In this section, the mathematical model of the engagement kinematics is derived. Then the nonlinear dynamic model of the interceptor with thruster is used to develop the integrated guidance and control equations in the pitch plane. As mentioned in the previous section, the IGC system is considered in the homing phase and does not affect other flight phases. Then, the control goal of the paper is described.

### Engagement kinematics

**Figure 1:**
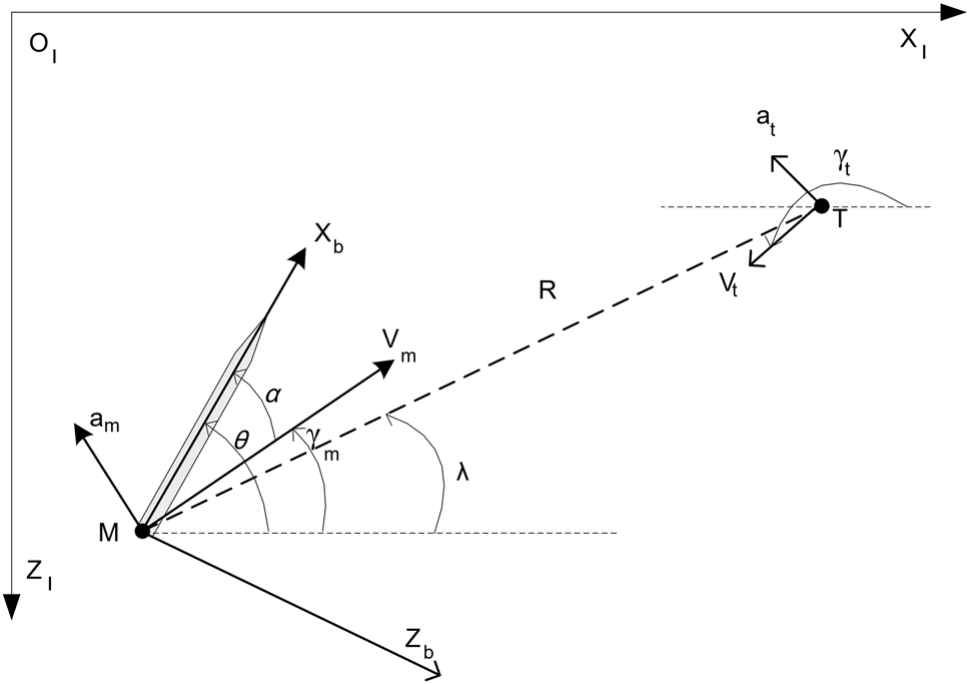
Planar engagement geometry.

The planar geometry of the missile and ballistic target engagement in the inertial system of $X_{I}$ - $O_{I}$ - $Z_{I}$ is shown in Fig. 1, where the missile and target are denoted by *M* and *T*, respectively. $V_t$ and $V_m$ are target and missile velocities and $\gamma _t$ and $\gamma _m$ are flight path angles, respectively. Also $a_t$ and $a_m$ are normal accelerations. In addition, *R* is the relative distance, and $\lambda$ is the line-of-sight (LOS) angle.

The missile-target relative motion kinematic model is established as^22^:

1 $$\begin{aligned}{}&\mathrm{I}.{} & {} \ {\dot{R}}= -V_t \cos (\gamma _t-\pi -\lambda )-V_m \cos (\gamma _m-\lambda ) \\ {}{} & {} {}&\ = V_t \cos (\gamma _t-\lambda )-V_m \cos (\gamma _m-\lambda ),\\&\mathrm{II}.{} & {} \ R{\dot{\lambda }}= -V_m \sin (\gamma _m-\lambda )-V_t \sin (\gamma _t-\pi -\lambda ) \\ {}{} & {} {}&\ = V_t \sin (\gamma _t-\lambda )-V_m \sin (\gamma _m-\lambda ),\\&\mathrm{III}.{} & {} \ {\dot{\gamma }}_{m}=a_m/V_m,\\&\mathrm{IV}.{} & {} \ {\dot{\gamma }}_{t}=a_t/V_t, \end{aligned}$$

where $V_m$ is assumed as constant, i.e., ${\dot{V}}_m=0$, and differentiating (1-$\mathrm{II}$) with respect to time and considering (1-$\mathrm{I}$)-(1-$\mathrm{IV}$), yield:

2 $$\begin{aligned} \ddot{\lambda }=-2 \frac{{\dot{R}}}{R} {\dot{\lambda }}+\frac{a_t \cos (\gamma _t-\lambda )}{R} -\frac{a_m \cos (\gamma _m-\lambda )}{R}\\+\frac{{\dot{V}}_t \sin (\gamma _t-\lambda )}{R} \end{aligned}$$

#### Remark 1

The term $a_t \cos (\gamma _t-\lambda )$ is the acceleration perpendicular to the target’s line-of-sight. Tactical ballistic targets generally do not have course correction maneuvers or escape maneuvers in the reentry phase, but even these targets can have accelerations due to the presence of fin installation errors^24^.

#### Remark 2

The term ${\dot{V}}_t \sin (\gamma _t-\lambda )$ is not considered in most studies in the terminal phase due to low drag, but it should be considered for ballistic targets in equations because of high velocity and high drag. For example, it can be seen in the simulation section that the speed of a tactical ballistic target in the final phase is about 2.7 times that of the interceptor, as a result, its drag force will be about 8 times, and it will be important to consider the drag acceleration even in a short time. However, if the velocity vector of these targets is not in line with the line-of-sight (which is not in most scenarios), a fraction of drag acceleration perpendicular to LOS will be projected, which makes complex near-miss engagement. For this reason, estimating this acceleration and its compensation in the interceptor guidance, significantly affects engagement success. In this study, the observer, estimates both acceleration terms perpendicular to LOS (due to maneuver and drag), which is used in the IGC law.

### Nonlinear dynamic model

**Figure 2:**
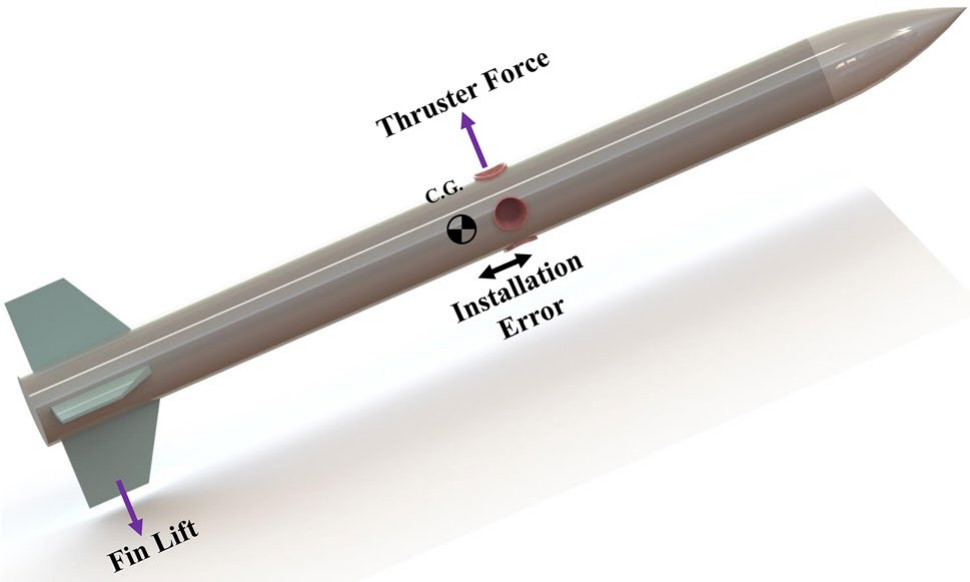
Interceptor with DCS thruster and fins.

The nonlinear model of the interceptor with ACS thruster has been presented in^25^. The model of the interceptor with tail fin and DCS thrusters (as shown in Fig. 2) is derived based on this model:

3 $$\begin{aligned}{\dot{\alpha }}&=\omega _y-F_z/mV \\ &{\dot{\omega }}_y=M_y/I_{YY}\\ &\alpha =\theta -\gamma \\ &{\dot{\theta }}=\omega _y \end{aligned}$$

where $\alpha$ is the angle of attack, $\omega _y$ denotes the pitch rate, $\theta$ is the pitch angle, *m*, $I_{YY}$ are the missile mass and pitch moment of inertia, and $F_z$, $M_y$ denote the lift force and the pitch moment, respectively. The corresponding expressions are:

4 $$\begin{aligned} &F_z=qS(C_{z_{\alpha }}\alpha + {C_{z_{\delta }}\delta }) +F_{th}+mg\cos (\gamma _m) \\&M_y=qSd(C_{m_{\alpha }}\alpha +C_{m_{\omega _y}}\frac{d}{2V} \omega _y+C_{m_{\delta }} \delta ) \end{aligned}$$

where *q* is the dynamic pressure, *S* is the aerodynamic reference area, *d* is the reference length, $F_{th}$ is the thruster force, $C_{z_{\alpha }}$ and $C_{z_{\delta }}$ are the lift force derivatives with respect to $\alpha$ and $\delta$. Also $\delta$ is the deflection angle for pitch control and $C_{m_{\alpha }}$, $C_{m_{\delta }}$ and $C_{m_{\omega _y}}$ are the pitch moment derivatives with respect to $\alpha$, $\delta$ and $\omega _y$, respectively.

Considering Eqs. (2) to (4) and defining $x_1={\dot{\lambda }}$, $x_2=\alpha$, $x_3= \omega _y$, $u_1=F_{th}$, $u_2=\delta$, the integrated model can be achieved as follows:

5 $$\begin{aligned} &{\dot{x}}_1=f_1 (x_1)+b_1 x_2+b_1^{'} u_1+d_1 \\&{\dot{x}}_2=f_2 (x_2,u_1)+b_2 x_3+d_2 \\&{\dot{x}}_3=f_3 (x_2,x_3)+b_3 u_2+d_3 \end{aligned}$$

where

6 $$\begin{aligned}{}&f_1(x_1)=-2\frac{{\dot{R}}}{R}{\dot{\lambda }} +\frac{g\cos (\gamma _m-\lambda )}{R}\cos (\gamma _m)\\&f_2 (x_2,u_1 )=(\frac{qSC_{z_{\alpha }}}{m V_m}\alpha +\frac{F_{th}}{m V_m}) \cos (\alpha )+\frac{g\cos (\gamma _m)}{V_m} \\&f_3 (x_2,x_3 )=\frac{qSdC_{m_{\alpha }}}{I_{YY}}\alpha +\frac{qSd^{2} C_{m_{\omega _y }}}{2I_{YY} V_m} \omega _y \end{aligned}$$

and

7 $$\begin{aligned}{}&b_1= \frac{qSC_{z_{\alpha }}}{m R} \cos (\gamma _m-\lambda ),b_1^{'}= \frac{1}{m R} \cos (\gamma _m-\lambda ) \\&b_2=1 \\&b_3=\frac{qSdC_{m_{\delta }}}{I_{YY }}\\&d_1= \frac{a_t \cos (\gamma _t-\lambda )}{R}+\frac{{\dot{V}}_t \sin (\gamma _t-\lambda )}{R}\\&d_2=d_2 (C_{z_{\alpha }},\Delta _2)\\&d_3=d_3 (C_{m_{\alpha }},C_{m_{\omega _y}},\Delta _3)+\frac{(x_{cg}-x_{thruster} ) F_{th}}{I_{YY}} \end{aligned}$$

#### Assumption 1

The term $C_{z \delta }$ in Eq. (4) is neglected because of the low lift of control tails compared to the body lift.

#### Assumption 2

Both the actuator and thruster have the bounds of $({\underline{\delta }},{\overline{\delta }})$ and $(\underline{F_{th}},\overline{F_{th}})$, because of the physical limitation of the actuator and pressure limitation in the gas generator of the thruster.

#### Assumption 3

In the terminal phase of engagement, ${\dot{R}}$ (by using the seeker data) and *R* (by using the fusion of seeker and radar data) are provided with acceptable accuracy. Also, the condition of successful engagement is that *R* is in the range of [0.1, 1] m.

#### Remark 3

In most references such as^18,22^, the term $\cos (\alpha )$ is not considered in Eq. (6). This does not seem right because the equation is derived with the assumption of acceleration being perpendicular to the velocity vector, and the acceleration due to $C_{z \alpha }$ and thruster are perpendicular to the body *x*-axis.

It is observed that the system of Eq. (5) is in the feedback form with mismatched uncertainty. These uncertainties are variable with time and functions of state variables. Also, $d_1$ is the acceleration perpendicular to the target$'$s LOS due to drag or maneuver, while $d_2$ and $d_3$ represent the time-varying perturbations caused by variations of aerodynamic parameters and external disturbances $\Delta _i$.

### Thruster description

**Figure 3:**
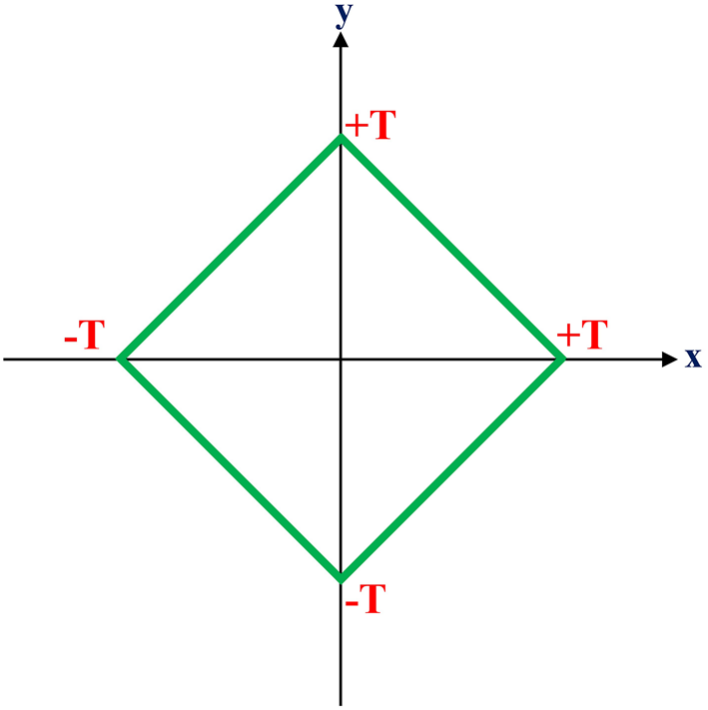
Feasible 2D thrust range with 4 nozzles DCS.

Thrusters are typically used in two situations: (a) Attitude Control System (ACS) thruster with less force at a specific distance from the center of mass; This case aims to apply the torque produced by thrusters and rapid rotation of the interceptor to capture the Angle Of Attack (AOA) and, as a result, produce lift force to increase acceleration in the desired direction. In this method, by increasing the altitude, air density decreases and both affect the interceptor’s acceleration. (b) DCS thruster with a greater center of mass force; This case aims to generate acceleration in the desired direction for a given period of time. In this class, the thruster acceleration will be independent of altitude. The interceptor with a thruster with four nozzles and the maximum force of T on the center of mass can generate an acceleration in a square area by controlling the valves of each nozzle, as shown in Fig. 3.

In this paper, it is assumed that the thruster computer can generate the arbitrary controller force in the pitch plane by changing the valves of its nozzle with good resolution in place, and there is no need for the discretization of the controller’s output command. The operation time of a thruster is 1*s* before the intercept, and it is activated by estimating $t_{go}$.

### IGC law design objective

In this study, the goal is to design an IGC law such that, subject to system (5), a miss distance less than 1 m is achieved against tactical ballistic target. For this purpose, the IGC law should guarantee that the state variable ${\dot{\lambda }} (x_1)$ is kept close to zero at the end of the terminal phase. Also, one should not be concerned about disturbance $d_1$ becoming infinity because, as the distance between the interceptor and target decreases to lower than 0.1 m, the simulation is stopped, and destruction occurs in this situation in reality.

## IGC law design with reduced order ESO

It is not possible to apply the classical ADRC structure so that all uncertainties are estimated using an ESO filter and then the estimated values are compensated to nullify LOS rate. The existence of mismatched uncertainty and the feedback form structure of the interceptor equation with two inputs, which differs fundamentally from the classical ADRC, are the root causes of this issue. Therefore, a novel concept is employed to deal with these equations by employing the ADRC concept and the framework for dealing with mismatched equations. The observer is created for each category of equations in this technique, which is identical to the ones used in^18,20^. The IGC law in this study is designed using the back-stepping structure and the RESO observer.

### Back-stepping based IGC law

A seeker is used to measure the relative parameters of the target and the interceptor. The input of equations defined in the first stage is $x_{1_d}=0$. Below are three typical modes:

1. The thruster input $(u_1)$ is considered zero in this mode and the control structure works to achieve the desired deflection since the thruster activation time corresponding to $t_{go}$ has not yet occurred. In this mode, the first Eq. in (5) changes as follows: ${\dot{x}}_1=f_1 (x_1)+b_1 x_2+d_1$.
2. The thruster is activated. In this instance, the thruster is initially responsible for providing $x_1^c$ (noting that the thruster time constant is less than fin actuator’s one). The deflection command is regarded as zero and the controller structure modifies the thruster force and direction until $x_1$ approaches the required value if the computed thrust force is less than the maximum thrust value.
3. The thruster is activated and the needed thrust force exceeds the thruster’s maximum force. In this situation, the angle of attack, which is determined by $x_{2}^c$, is responsible for supplying the necessary acceleration difference. The required values to compute the reference command of angular velocity are obtained by sending $x_{2}^c$ through a differentiation filter. Subsequently, the necessary deflection command is given using $x_{3}^c$ and $\dot{x_{3}^c}$.

All these phases assume that the controller has access to ${\hat{d}}_i$ (the estimation of $d_i$) instead of $d_i$, which is the output of RESO filter with appropriate bandwidth. In the following, the design of the IGC law for the first mode is completely done, and then with the addition of the thruster and use of the daisy chain method, this process is developed for the third mode. The second mode does not require revision because it is only a particular instance of the third mode.

### Step1

The purpose of IGC is zeroing LOS rate (${\dot{\lambda }}$), that is, $x_1^c=0$.

Equation (8) shows the expected dynamics to meet this demand:

8 $$\begin{aligned} {\dot{x}}_1= -k_1 x_1,k_1>0 \end{aligned}$$

where $k_1$ is the controller coefficient and indicates the convergence speed to the origin.

The first dynamic surface is defined as:

9 $$\begin{aligned} z_1=x_1-x_1^c=x_1-0 \end{aligned}$$

Differentiating (9) with respect to time provides:

10 $$\begin{aligned} &{\dot{z}}_1= f_1 (x_1 )+b_1 x_2+{\hat{d}}_1 \\&{\dot{z}}_1= f_1 (x_1 )+b_1 x_2^c+{\hat{d}}_1+b_1 (x_2-x_2^c) \end{aligned}$$

where ${\hat{d}}_1$ is the estimation of $d_1$. The dynamics (8) may then be obtained by determining $x_2^c$ as a virtual command in the form of (11) using the inverse dynamics method.

11 $$\begin{aligned} x_2^c=b_1^{-1} \left( -f_1 (x_1 )-{\hat{d}}_1-k_1 z_1\right) \end{aligned}$$

The objective of the second phase is to choose $x_3^c$ such that the the $x_2$ state, or the angle of attack ($\alpha$), tracks the command value. This calls for altering the dynamics of the angle of attack, as follows:

12 $$\begin{aligned} {\dot{\alpha }}={\dot{\alpha }}_c-k_2 (\alpha -\alpha _c ),k_2>0 \end{aligned}$$

where Eq. (10) is used to calculate $\alpha _c$. A differentiator filter is used to compute ${\dot{\alpha }}_c$ as follows:

13 $$\begin{aligned} G(s)=\frac{\omega s}{s+\omega } \end{aligned}$$

where $\omega$ is the bandwidth of the derivative filter and $``s''$ is the Laplace notation. Filter (13) is used to prevent the explosion of complexity in the analytical calculation. For this computation, there are different approaches, such as using the command filter^26^. Due to the short duration of the final phase and the presence of a thruster, it is not required to employ these methodologies in this study.

By defining the second dynamic surface as:

14 $$\begin{aligned} z_2=x_2-x_2^c \end{aligned}$$

and differentiating it with respect to time, we have:

15 $$\begin{aligned} &{\dot{z}}_2={\dot{x}}_2-{\dot{x}}_2^c \\&\,\,\,\,\,\,\,=f_2 (x_2 )+ b_2 x_3^c+{\hat{d}}_2-{\dot{x}}_2^c+b_2 (x_3-x_3^c) \end{aligned}$$

where ${\hat{d}}_2$ is the estimation of $d_2$. Now, to achieve dynamics in (12), it is suggested to choose the virtual command $x_3^c$ as follows:

16 $$\begin{aligned} x_3^c=b_2^{-1} \left( -f_2 (x_2 )-{\hat{d}}_2+{\dot{x}}_2^c-k_2 z_2\right) \end{aligned}$$

The controller design without thruster mode is completed after the pitch rate achieves the command value utilizing the suitable elevator. For this reason, the third dynamic surface was defined in the final step as follows:

17 $$\begin{aligned} z_3=x_3-x_3^c \end{aligned}$$

By differentiating (17) with respect to time, together with (14) yields:

18 $$\begin{aligned} {\dot{z}}_3={\dot{x}}_3-{\dot{x}}_3^c=f_3 (x_2,x_3 )+ b_3 u_2+{\hat{d}}_3-{\dot{x}}_3^c \end{aligned}$$

where ${\hat{d}}_3$ is the estimation of $d_3$. Stable dynamics (20) is generated to send $\omega _y$ to $\omega _y^c$ by passing $x_3^c$ through (13) and computing the elevator input as follows:

19 $$\begin{aligned} u_2=b_3^{-1} \left( -f_3 (x_2,x_3 )-{\hat{d}}_3+{\dot{x}}_3^c-k_3 z_3 \right) , k_3>0 \end{aligned}$$

20 $$\begin{aligned} {\dot{\omega }}_y={\dot{\omega }}_y^c-k_3\left( \omega _y-\omega _y^c\right) \end{aligned}$$

The design created for the first mode is now modified by using the thruster with daisy chain method. The first option in this situation will be to zero the LOS rate by thruster. Since it has a shorter time constant than the electromechanical actuator and does not encounter the rotation rate limit of electromechanical actuators. Then, the difference between the maximum thruster acceleration and the command acceleration is provided by the angle of attack $(x_2)$. Consequently, Eqs. (10) and (11) are converted to forms (21) and (22), respectively:

21 $$\begin{aligned}{}&{\dot{z}}_1=f_1 (x_1 )+b_1 x_2+b_1^{'} u_1+{\hat{d}}_1 \\&u_1={b_1^{'}}^{-1}\left( -f_1 (x_1 )-{\hat{d}}_1-b_1 x_2-k_1 z_1\right) \end{aligned}$$

22 $$\begin{aligned} x_2^c=\frac{u_1-\max {(F_{th})}}{qSC_{z_{\alpha }}} \end{aligned}$$

From this stage on, the definition of $z_2$ is continued as previous. The angle of attack command and therefore the elevator command will be zero if the necessary acceleration is less than the thruster’s maximum acceleration.

It is worth noting that in this disturbance rejection-based control structure, $\alpha _c$ functions as the guidance loop’s output while $\omega _y^c$ acts as the controller’s inner loop command. As mentioned in reference^22^, when this control structure is used, the advantage of IGC compared to conventional guidance and control is lost. However, it should be noted in this structure, the dynamics of the interceptor is still considered in the calculation of the guidance command. This differs from the conventional separate designs for the guidance and control loops, which take the dynamics of the interceptor as a point mass in guidance loop. This issue performs better in high-speed engagement geometry changes. Then, the inherent problem in the classical IGC approach should be considered, which ignores the interceptor’s inherent longitudinal and angular dynamics difference, as was mentioned in^27^, and led to the development of the partial IGC approach with a structure similar to that used in this study.

### Observer design

It was assumed in the previous part that ${\hat{d}}_i$ is provided as the estimator’s output. A variety of concepts can be applied while designing the estimator, including ESO, Super twisting ESO, Reduced-order ESO, and high-order nonlinear filters. Each of these approaches has its specific benefit; for example, using super-twisting ESO can ensure the filter’s convergence in a finite-time^19^ or using high-order nonlinear filters can improve performance when predicting high-frequency disturbances. The RESO estimator, which according to^18^ has a greater bandwidth than the ESO, is utilized in this study because of the high speed terminal phase and the lack of high-frequency disturbances. This section reviews how to implement this approach by designing reduced-order ESO as referred in^28^. Since $d_i$ is present in the dynamics of all three state variables, an ESO estimator like the classical ADRC cannot be used to estimate the total disturbances; instead, this filter needs to be created independently for each state variable.

#### Assumption 4

The constant positive values ${\overline{d}}_1$, ${\overline{d}}_2$, ${\overline{d}}_3$ exist for uncertainty of the system (5) such that $d_1$, $d_2$, $d_3$ meets $|d^r d_i/dt^r |\le {\overline{d}}_i$,$(i=1-3,r=0,1)$ , i.e. the disturbances and their derivatives are all bounded.

For example, concerning the guidance loop, RESO is as follows:

23 $$\begin{aligned} \left\{ \begin{array}{l} {\dot{p}}_1= -\beta _1 p_1-\beta _1^2 x_1-\beta _1 \left( f_1 (x_1 )+b_1 x_2+b_1^{'} u_1\right) \\ {\hat{d}}_1=p_1+\beta _1 x_1, \beta _1>0 \\ \end{array} \right. \end{aligned}$$

where $p_1$, $\beta _1$ and ${\hat{d}}_1$ represent the filter variable, filter bandwidth, and estimated disturbances in the guidance loop, respectively. In addition, the same procedure is repeated for estimating ${\hat{d}}_2$ and ${\hat{d}}_3$, as in Eqs. (24) and (25) below:

24 $$\begin{aligned} \left\{ \begin{array}{l} {\dot{p}}_2= -\beta _2 p_2-\beta _2^2 x_2-\beta _2 \left( f_2 (x_2,u_1 )+b_2 x_3\right) \\ {\hat{d}}_2=p_2+\beta _2 x_2, \beta _2>0 \\ \end{array} \right. \end{aligned}$$

25 $$\begin{aligned} \left\{ \begin{array}{l} {\dot{p}}_3= -\beta _3 p_3-\beta _3^2 x_3-\beta _3 \left( f_3 (x_2,x_3 )+b_3 u_2\right) \\ {\hat{d}}_3=p_3+\beta _3 x_3, \beta _3>0 \\ \end{array} \right. \end{aligned}$$

As $\beta$ increases, the observer bandwidth also increases. Practically, the bandwidth cannot be extended to the intended level due to actuator and sensor data acquisition delays, as well as noise on the sensors. Additionally, the estimation error can be decreased by raising $\beta$. Theorem 1 states the relationship between estimation error and $\beta$ values.

#### Theorem 1

Considering the observer designed in (23) to (25) and Assumption 4 for system (5), we have:

26 $$\begin{aligned} \left\| {\textbf {E}}_0\right\| \le \frac{\max {({\overline{d}}_i})}{\min {(|\beta _i|)}}, i=1,2,3 \end{aligned}$$

where $${\textbf {E}}_0={[d_1-{\hat{d}}_1,d_2-{\hat{d}}_2,d_3-{\hat{d}}_3]}^T$$.

#### Proof

This theorem has been proved in detail in^18^. It can also be proved using comparison lemma.

27 $$\begin{aligned} e_{o_1} & =d_1-{\hat{d}}_1\Longrightarrow {\dot{e}}_{o_1}={\dot{d}}_1-({\dot{p}}_1+\beta _1 {\dot{x}}_1 ) \\&={\dot{d}}_1+\beta _1 p_1+\beta _1^2 x_1+\beta _1 \left( f_1 (x_1 )+b_1 x_2+b_1^{'} F_{th} \right) \\& \quad -\beta _1 \left( f_1 (x_1 )+b_1 x_2+b_1^{'} F_{th}+d_1 \right) \\&={\dot{d}}_1+\beta _1 p_1+\beta _1^2 x_1-\beta _1 d_1 \\&={\dot{d}}_1+\beta _1 ({\hat{d}}_1-d_1 )={\dot{d}}_1-\beta _1 e_{o_1} \end{aligned}$$

By using the comparison lemma, the following inequality is obtained:

28 $$\begin{aligned} |e_{o_1}|\le \underset{t \ge 0}{\max }\left( \frac{|{\dot{d}}_1(t)|}{\beta _1}\right) \left( 1-e^{-\beta _1 t} \right) +d_1 e^{-\beta _1 t} \end{aligned}$$

such that the error of observer is bounded for all $t\ge 0$.

Similarly, convergence of other RESO observers (which estimate ${\hat{d}}_2$ and ${\hat{d}}_3$) can be proved. 

#### Remark 4

It should be emphasized that the estimation error will be asymptotically stable by utilizing the observer (23) and adequate adjustment of $\beta$ if the magnitude of disturbance is constant, i.e. ${\dot{d}}_i=0$.

### Gain tuning

The bandwidth of the RESO is equal to $\beta$. As $\beta$ increases, the speed of disturbance estimation also increases. Due to the noise of the sensors and also the delay in the system, this value cannot be increased arbitrarily in practice. Controller gains ($k_1$, $k_2$ and $k_3$), indicate the speed of convergence for LOS rate and tracking for $\alpha$ and *q*. Also, due to the use of a cascade structure in the control law, the bandwidth of the inner loop must be faster than the middle loop and the outer loop, in order to achieve the desired performance. Here too, the bandwidth of the actuator in practice, makes it impossible to speed up the tracking as much as desired. With these explanations, the following method can be used for the initial gains tuning. First, gain $k_3$ (which represents the bandwidth of the inner loop) is chosen a little less than the bandwidth of the actuator, and then we choose the bandwidth of the middle and outer loops, respectively, about 2 to 5 times smaller than the previous loop. After that, we should determine the observer’s gains. In this regard, the observer gain related to each loop can be selected from 2 to 10 times its control bandwidth, depending on the rate of disturbance changes in that loop. It should be kept in mind that the lack of a sufficient difference between the bandwidth of the inner loop and the actuator or the outer loops can lead to instability or bad tracking.

## Stability analysis of the closed-loop system

In this section, the stability of the closed loop system is investigated using Lyapunov theorem.

### Theorem 2

Consider the IGC system (5), if Assumption 4 and Theorem 1 are satisfied under the condition that the control gain and observer bandwidth satisfy $k_i>0$, $\beta _i>0$, $i=1,2,3$, there exists a positive value for $k_i$ such that following nonlinear IGC law (29) combined with the RESO estimator can guarantee that the tracking error converges to the origin asymptotically.

29 $$\begin{aligned} \left\{ \begin{array}{l} x_{2_{thrust}}={b_1^{'}}^{-1} \left( -k_1 x_1-f_1 (x_1 )-{\hat{d}}_1 \right) \\ F_{th}=F_{th_{max}}\times \mathrm{sign} (x_{2_{thrust}} )\times \min \left( \frac{|x_{2_{thrust}}|}{F_{th_{max}}},1\right) \\ x_{2_{aero}}=b_1^{'} b_1^{-1} \left( x_{2_{thrust}}-F_{th} \right) \\ x_{3_c}=b_2^{-1} \left( {\dot{x}}_{2_{aero}}-k_2 \left( x_2-x_{2_{aero}}\right) -f_2 (x_2 )-{\hat{d}}_2 \right) \\ u=b_3^{-1} \left( {\dot{x}}_{3_c}-k_3 \left( x_3-x_{3_c}\right) -f_3 (x_3 )-{\hat{d}}_3 \right) \\ \end{array} \right. \end{aligned}$$

### Proof

In the first step, consider the tracking error as follows:

30 $$\begin{aligned} {\textbf {E}}= \begin{bmatrix} e_1 \\ e_2 \\ e_3 \end{bmatrix} = \begin{bmatrix} x_1-0 \\ x_2-x_{2_{{aero}}} \\ x_3-x_{3_c} \end{bmatrix} \end{aligned}$$

31 $$\begin{aligned} \Longrightarrow \dot{{\textbf {E}}}&= \begin{bmatrix} {\dot{x}}_1 \\ {\dot{x}}_2-{\dot{x}}_{2_{{aero}}} \\ {\dot{x}}_3-{\dot{x}}_{3_c} \end{bmatrix}\\&=\begin{bmatrix} {f_1 (x_1 )+b_1 \left( x_{2_{aero}}+e_2\right) +b_1^{'}F_{th}+d_1} \\ {f_2 (x_2 )+b_2 (e_3+x_{3_c })+d_2-{\dot{x}}_{2_{aero}}} \\ {f_3 (x_3 )+b_3 u+d_3-{\dot{x}}_{3_c}} \end{bmatrix}\\&={ \begin{bmatrix} f_1 (x_1)+b_1 (x_{2_{aero}}+e_2 )+b_1^{'} x_{2_{thrust}}-b_1 x_{2_{aero}}+d_1 \\ f_2 (x_2 )+b_2 e_3+{\dot{x}}_{2_{aero}}-k_2 \left( x_2-x_{2_{aero}} \right) -f_2 (x_2)-{\hat{d}}_2+d_2-{\dot{x}}_{2_{aero}} \\ f_3 (x_3 )+{\dot{x}}_{3_c}-k_3 \left( x_3-x_{3_c })-f_3 (x_3 \right) -{\hat{d}}_3+d_3-{\dot{x}}_{3_c} \end{bmatrix}}\\&= \small {\begin{bmatrix} f_1 (x_1 )+b_1 e_2+b_1^{'} \left( {b_1^{'}}^{-1} \left( -k_1 x_1-f_1 (x_1 )-{\hat{d}}_1 \right) \right) +d_1 \\ b_2 e_3-k_2 \left( x_2-x_{2_{aero}} \right) -{\hat{d}}_2+d_2 \\ -k_3 (x_3-x_{3_c } )-{\hat{d}}_3+d_3 \end{bmatrix}}\\&= \begin{bmatrix} b_1 e_2-k_1 e_1+e_{o_1} \\ b_2 e_3-k_2 e_2+e_{o_2} \\ -k_3 e_3+e_{o_3} \end{bmatrix}\\ \end{aligned}$$

Consider the following Lyapunov function:

32 $$\begin{aligned} V&=\frac{1}{2} {\textbf {E}}^T {\textbf {E}} \\&{\dot{V}}={\textbf {E}}^T \dot{{\textbf {E}}}={\textbf {E}}^T\left( {\begin{bmatrix} b_1e_2-k_1e_1+e_{o_1} \\ b_2e_3-k_2e_2+e_{o_2} \\ -k_3e_3 + e_{o_3} \end{bmatrix}} {\textbf {E}} + \begin{bmatrix} e_{o_1} \\ e_{o_2} \\ e_{o_3} \end{bmatrix} \right) \\ {}&\ = {\textbf {E}}^T \begin{bmatrix} -k_1 &{} b_1 &{} 0 \\ 0 &{} -k_2 &{} b_2 \\ 0 &{} 0 &{} -k_3 \end{bmatrix} {\textbf {E}} + {\textbf {E}}^T \begin{bmatrix} e_{o_1} \\ e_{o_2} \\ e_{o_3} \end{bmatrix} \\ {}&{\ = {\textbf {E}}^T {\begin{bmatrix} -k_1 &{} \frac{b_1}{2} &{} 0 \\ \frac{b_1}{2} &{} -k_2 &{} \frac{b_2}{2} \\ 0 &{} \frac{b_2}{2} &{} -k_3 \end{bmatrix}} {\textbf {E}} + {\textbf {E}}^T \begin{bmatrix} e_{o_1} \\ e_{o_2} \\ e_{o_3} \end{bmatrix} {\ = {\textbf {E}}^T{\textbf {P}}{} {\textbf {E}}} + {\textbf {E}}^T \begin{bmatrix} e_{o_1} \\ e_{o_2} \\ e_{o_3} \end{bmatrix}} \end{aligned}$$

${\textbf {E}}_o$ is defined by,

33 $$\begin{aligned} {\textbf {E}}_o= \begin{bmatrix} e_{o_1} \\ e_{o_2} \\ e_{o_3} \end{bmatrix} \end{aligned}$$

Matrix ${\textbf {P}}$ is defined by,

34 $$\begin{aligned} {\textbf {P}}= \begin{bmatrix} -k_1 &{} \frac{b_1}{2} &{} 0\\ \frac{b_1}{2} &{} -k_2 &{} \frac{b_2}{2} \\ 0 &{} \frac{b_2}{2} &{} -k3 \end{bmatrix} \end{aligned}$$

By assuming $k_1$, $k_2$, and $k_3$ as positive gains which satisfy following inequalities, it can be deduced that matrix ${\textbf {P}}$ is negative-definite,

35 $$\begin{aligned} \left\{ \begin{array}{l} \frac{b_1^2}{4}<k_1 k_2 \\ \frac{b_2^2}{4}<k_2 k_3 \\ k_1, k_2,k_3>0\\ \end{array} \right. \end{aligned}$$

Keeping in mind, the statement that a matrix is positive (negative) definite if and only if all of its principal minors are positive (negative), the latter is concluded. Now the gains can be tuned as follows:

36 $$\begin{aligned} \left\{ \begin{array}{l} k_1=\frac{b_1^2}{4c}+r \\ k_2=c+r \hspace{4mm},\hspace{20mm} r,c > 0 \\ k_3=\frac{b_2^2}{4c}+r\\ \end{array} \right. \end{aligned}$$

Note that *c* and *r* can be set arbitrarily. By using such gains, it can be verified easily that ${\textbf {P}}$ satisfies following matrix inequality:

37 $$\begin{aligned} {\textbf {P}}\le -r{\textbf {I}}<0 \end{aligned}$$

Hence, ${\dot{V}}$ is bounded, and its upper bound is given by,

38 $$\begin{aligned} {\dot{V}}\le {\textbf {E}}^T (-r{\textbf {I}}){\textbf {E}}+{\textbf {E}}^T {\textbf {E}}_o\le - \left\| {\textbf {E}}\right\| (r\left\| {\textbf {E}}\right\| -\left\| {\textbf {E}}_o \right\| ) \end{aligned}$$

So ultimate bound of $\left\| {\textbf {E}}\right\|$ can be computed as follows,

39 $$\begin{aligned} \left\| {\textbf {E}}\right\| \le \frac{\left\| {\textbf {E}}_o\right\| }{r}\le \frac{\sqrt{3}\max \left( |e_{o_1}|,|e_{o_2}|,|e_{o_3}|\right) }{r} \end{aligned}$$

## Seeker filter design

Guidance filter plays a crucial role in overall performance of an air defense system^29^. It is well-known that various error sources of onboard seeker, such as low sampling rate besides time-delayed and noisy measurements form the main challenges to achieve a hit-to-kill performance. However, according to authors’ knowledge, all existing IGC schemes have assumed an almost ideal guidance filter to utilize true LOS rate value as the measured variable. Keeping this in mind, a two-stage guidance filter is employed in this study to account for exact known engagement kinematics along with an accurate model of seeker error sources. The first filter stage is inspired by^30^ which is briefly explained in what follows.

Assume the update rate and measurement delay of seeker to be $T_s$ and $\tau _d$, respectively. Furthermore, the pointing angle and attributed LOS rate are expressed by $\epsilon (t)$ and $\omega (t)$, respectively. The following filter dynamics in pitch plane is given:

40 $$\begin{aligned} &\begin{bmatrix} {\dot{\epsilon }}(t) \\ {\dot{\omega }}(t) \end{bmatrix} =\begin{bmatrix} 0 &{} 1 \\ 0 &{} 0 \end{bmatrix} \begin{bmatrix} \epsilon (t) \\ \omega (t) \end{bmatrix} + \begin{bmatrix} -r(t) \\ 0 \end{bmatrix},\\&y(t)= \begin{bmatrix} 1&0 \end{bmatrix} \begin{bmatrix} \epsilon (t) \\ \omega (t) \end{bmatrix} \end{aligned}$$

in which *r*(*t*) denotes the inertial angular velocity of inner gimbal measured by gimbal’s gyroscope. The measurement equation indicates that pointing angle is directly measured by seeker. A classical discrete time filter with gains of $L_1$ and $L_2$ is applied to (40) as follows:

41 $$\begin{aligned} \begin{bmatrix} {\hat{\epsilon }}(t+T) \\ {\hat{\omega }}(t+T) \end{bmatrix} = \begin{bmatrix} 1 &{} T \\ 0 &{} 1 \end{bmatrix} \begin{bmatrix} {\hat{\epsilon }}(t) \\ {\hat{\omega }}(t) \end{bmatrix} - \begin{bmatrix} 1 \\ 0 \end{bmatrix} \psi (t+T,t)+...\\ \begin{bmatrix} L_1+\tau L_2 \\ L_2 \end{bmatrix} \begin{bmatrix} y(t+T)-{\overline{\epsilon }}(t+T-\tau ) \end{bmatrix}, \end{aligned}$$

where

42 $$\begin{aligned} \psi (t_2,t_1)=\int _{t_1}^{t_2} r(s)ds \end{aligned}$$

and

43 $$\begin{aligned} {\overline{\epsilon }}(t+T-\tau )={\hat{\epsilon }}(t)+(T-\tau ) {\hat{\omega }}(t)-\psi (t+T-\tau ,t). \end{aligned}$$

Utilizing the filter dynamics in (41) assures the independence of estimated LOS rates from the interceptor’s body angular motions as an important guidance filter performance index^29^. Following straightforward calculations, one can derive the estimated LOS rate dynamic as follows

44 $$\begin{aligned} {\hat{\omega }}(t)=\frac{L_2(T-\tau )z+\tau L_2}{z^2+(L_1+L_2 T-2)z+1-L_1} \omega (t) \end{aligned}$$

Denoting the standard deviation of pointing angle gaussian noise by $\sigma _n$, according to (44), the standard deviation of LOS rate estimation is achieved as follows:

45 $$\begin{aligned} \sigma _{\omega }^2=\frac{2L_2^2}{L_1 (4-2L_1-L_2 T)} \sigma _n^2 \end{aligned}$$

$L_1$ and $L_2$, are determined in a way which the closed-loop poles of (44) correspond to a standard second order continuous characteristic equation with natural frequency $\omega _c$ and damping ratio $\zeta _c$. It is nice to mention that accounting for range-dependent measurement noise for an active seeker, $\sigma _n$ shall preserve the following equality

46 $$\begin{aligned} \sigma _n=\sigma _0 \left( \frac{R_{TM}}{R_{TM_0}}\right) ^2 \end{aligned}$$

where $\sigma _0$ is the standard deviation of seeker angle measurement error which is a function of signal to noise ratio (SNR) and seeker beam width. Also, $R_{TM_0}$ is the distance between interceptor and target in the beginning of endgame phase. It is determined according to (46) the decrease of noise level as the relative range goes toward zero.

### Remark 5

The main idea behind this section, is to obtain a reasonable model to evaluate the effects of the seeker filter’s bandwidth and sensor’s measurement noise on proposed guidance law performance, as two significant practical issues. Evidently one can use more sophisticated filter schemes such as those introduced in^29,31,32^ to be implemented on seeker’s computer. After that, there is a second filter (23) in the interceptor’s computer that uses this LOS rate as input to estimate the target’s acceleration and uncertainty due to the aerodynamic coefficients.

## Simulation results

Numerous numerical simulations were performed, and the results are reported in this section to evaluate the performance of the proposed IGC law in conjunction with the thruster and to demonstrate its ability to intercept high-speed and accelerated targets. This section examines various aspects of this research through four hypothetical situations. All of these scenarios pertain to an interceptor’s terminal phase against a tactical ballistic target with a maximum velocity of 2500*m*/*s*. The angle between the interceptor’s velocity vector and the predicted intercept point (PIP) suggests that the mid-course guidance failed to nullify the zero effort miss (ZEM) at the beginning of the terminal phase. In this circumstance, it was assumed that:

47 $$\begin{aligned}{}[{\dot{\lambda }}(0),\alpha (0),\omega _y (0)]=[{\dot{\lambda }}_0,0,0] \hspace{3mm} \end{aligned}$$

The interceptor’s specifications are listed in Table 1.

**Table 1:** The interceptor properties in the terminal phase.

Parameter	Value
Mass	180 kg
Diameter	260 mm
$I_{YY}$	350 $kg.m^2$
$C_{z_\alpha }$	$-17$ $radian^{-1}$
$C_{m_\alpha }$	$-28$ $radian^{-1}$
$C_{m_\delta }$	$-28$ $radian^{-1}$

As mentioned above, the interceptor has an active seeker, and the terminal phase range is less than 7*km* due to issues such as the frequency band of the seeker, its limited power, and the RCS of ballistic targets in that band. Due to the high relative velocity, the homing phase range is highly effective for ZEM ability to compensate. In addition, $0.2^{\circ }$ was chosen as the standard deviation of the angle measurement error $(\sigma _0)$ for this range.

**Table 2:** The kinematic properties of the interceptor and the target at the start of the terminal phase.

Parameter	Value
Interceptor-target distance	7 km
Altitude	12 km
Target velocity	2500 m/s
Target maneuver	step 9 g
Target drag acceleration	8g $@t=t_0$
Interceptor velocity	900 m/s
$\lambda _0$	45$^{\circ }$
$\gamma _{m_0}$	47°
$\gamma _{t_0}$	225°
ZEM $@t=t_0$	60 m

The relative velocity along the LOS and the interceptor-target distance at each instant were utilized to calculate the time to go $(t_{go})$, and the thruster was activated just 1*s* before the interception. In addition, the thruster had a maximum acceleration of 8 g.

The bandwidth of the differentiator filter is assumed to be 15 rad/s, whereas the bandwidth of the RESO estimators (for all channels) are set as $\beta _1=10$, $\beta _2=20$ and $\beta _3=40$rad/s. In addition, the controller gains are selected as follows:

48 $$\begin{aligned} k_1=1,\ k_2=5,\ k_3=15 \end{aligned}$$

After passing through the second order transfer function (49), the elevator command was applied to the simulation by passing the rate limit block up to a maximum of $250^{\circ }/s$ and then the saturation block up to a maximum of $\pm 28^{\circ }$.

49 $$\begin{aligned} \frac{\delta }{\delta _c}=\frac{\omega _n^2}{s^2+2\zeta \omega _n s+\omega _n^2} \end{aligned}$$

where $\omega _n$ is actuator natural frequency and $\zeta$ is damping factor with $\omega _n=20Hz$, $\zeta =0.7$.

Notably, including the actuator rate limit in the simulation leads to make the case realistic and reduces the controller bandwidth, significantly impacting the engagement outcome. Simulations indicate that actuator rate must be increased for a proper engagement at higher altitudes. Design-wise, this rate should be increased by increasing the interceptor’s height and decreasing the hinge moment.

The interceptor was assumed to have a maximum structural load of 22 g due to aerodynamics and thrusters, leading to a restriction of $\alpha _c$.

Finally, miss distance with ZEM calculation is reported at $t_{go}=0$.

### Remark 6

To calculate ZEM according to reference^33^, the following formula is used:

50 $$\begin{aligned} ZEM = \frac{\left| \dot{{\textbf {R}}}\times {\textbf {R}} \right| }{\left| \dot{{\textbf {R}}} \right| } \end{aligned}$$

where $\dot{{\textbf {R}}}$ and ${\textbf {R}}$ are relative velocity vector and relative position vector in inertial frame, respectively.

### Case 1

**Figure 4:**
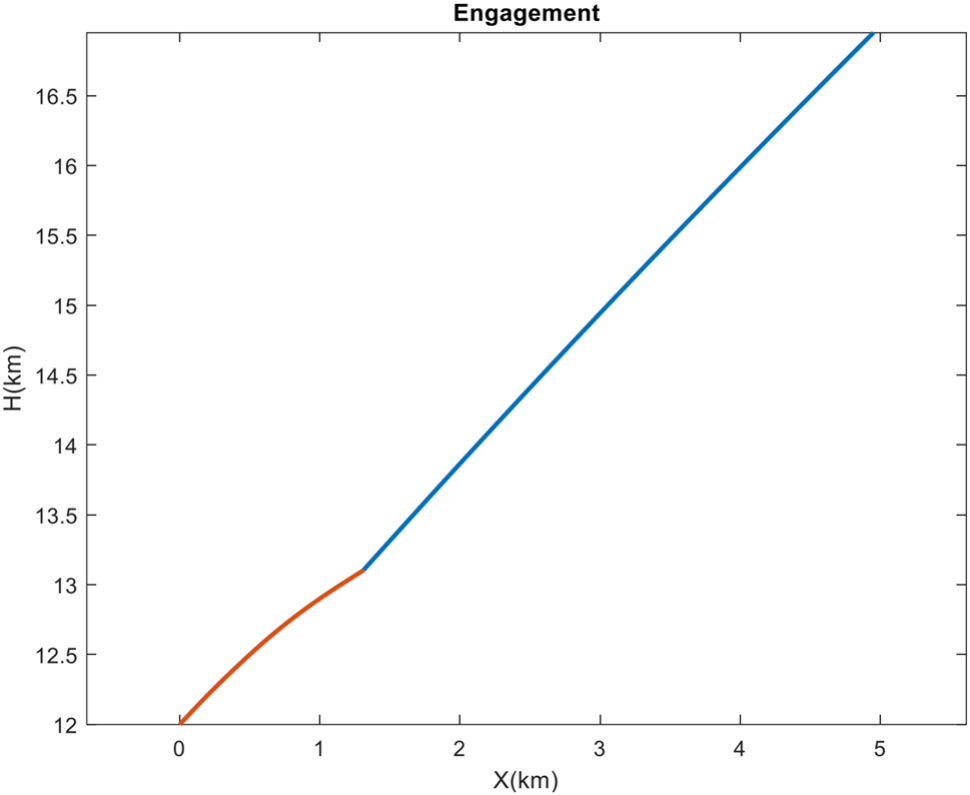
The engagement geometry in Case 1.

The objective of the first scenario is to engage a ballistic target with the specifications listed in Table 2. This scenario aimed to demonstrate the effectiveness of the proposed IGC method for a dual-controlled interceptor. The terminal phase is begun at an altitude of 12 km and ended at 13 km. The problem carried the following uncertainties and disturbances: 1. Acceleration of the 9g step of the target (as stated previously, the source of this acceleration could be an error in the installation of the ballistic warhead’s fins or the presence of a maneuver to change the trajectory of some tactical ballistic missiles); 2. An 8g drag acceleration at the beginning of the scenario; 3. Reduction of aerodynamic coefficients $C_{z_{\alpha }}$, $C_{m_{\alpha }}$, and $C_{m_{\delta }}$ in the controller relative to simulation equations by $30\%$, $25\%$ and $25\%$, respectively; 4. A 7 cm installation error between the thruster nozzle and the interceptor’s center of mass. ${\dot{\lambda }}_{true}$ was supplied directly to (10), (21) and (23) without passing through the filter and adding noise based on the ideal seeker assumption. The kinematics of the engagement is depicted in Figure 4.

**Figure 5:**
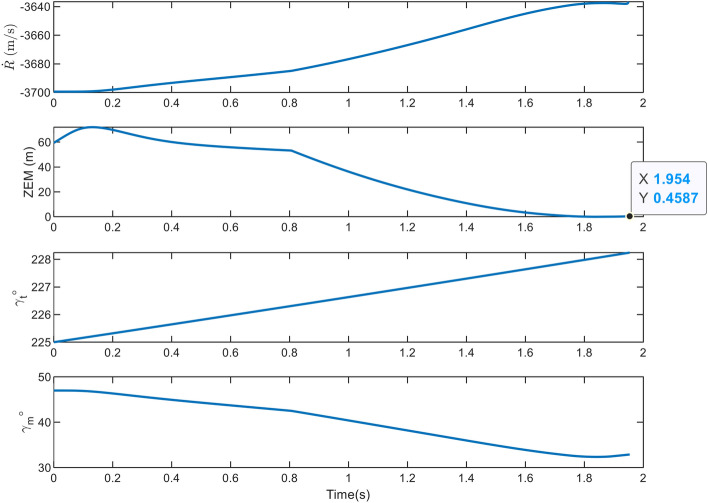
The changes in path angles, ZEM and relative velocity in case 1.

**Figure 6:**
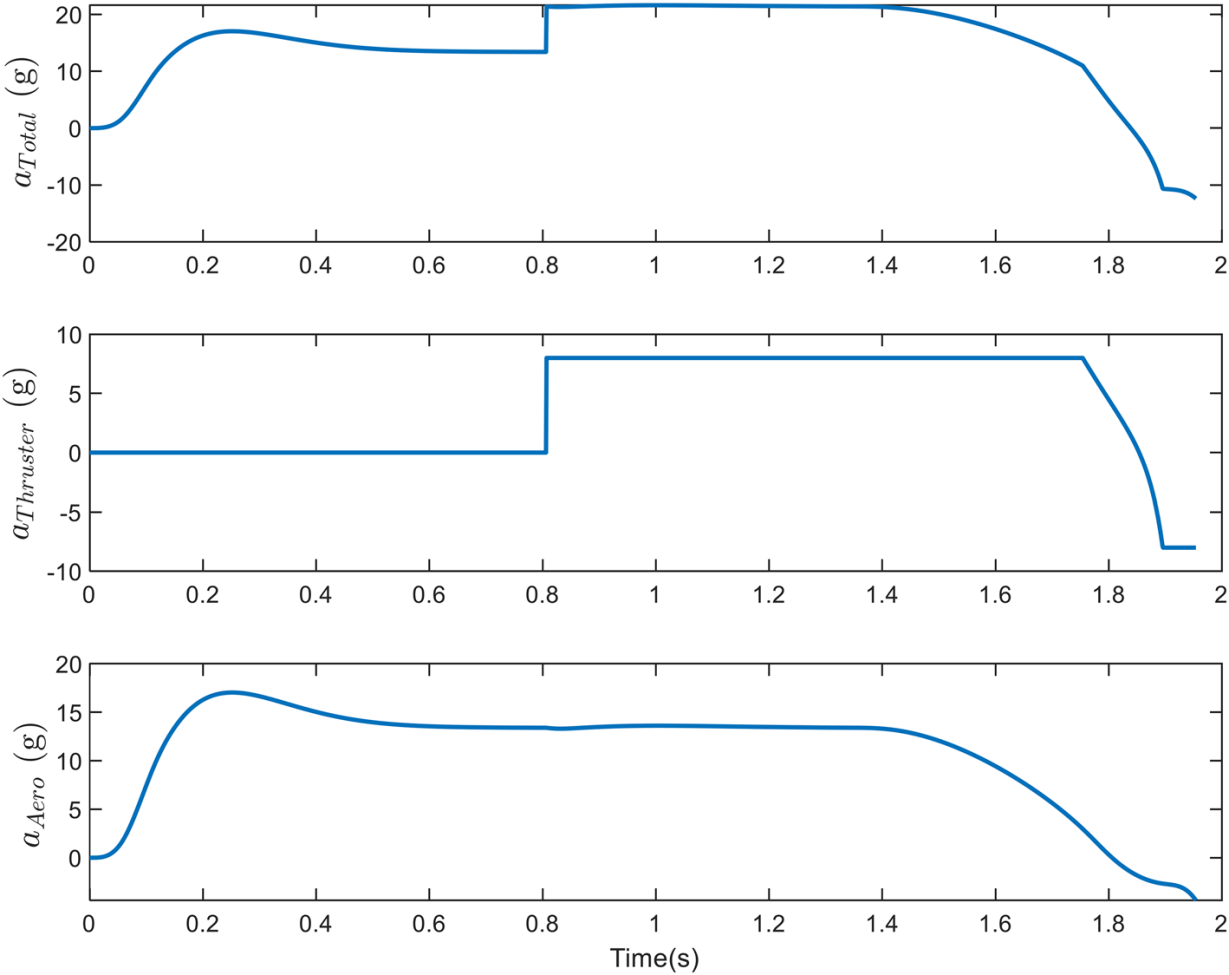
Acceleration of the interceptor due to the angle of attack, thruster activation and the total ones in case 1.

The final phase’s engagement time was 1.95*s*, and the obtained miss distance was 0.46 m, as represented in Figure 5. This value indicates the direct interception and success of the scenario. Due to the target’s high speed, the velocity vector has rotated approximately $3.5^{\circ }$ due to gravity, maneuver, and drag, which can result in a larger miss distance if the acceleration is not estimated and compensated for in the IGC law. As depicted in Figure 6, the interceptor’s velocity vector rotated by $14^{\circ }$ in a short period, causing a significant acceleration to compensate for the error. Before using the thruster, the interceptor utilized all its aerodynamic capabilities to minimize the error. The thruster was then activated 1*s* before the termination. As the error decreased, the aerodynamic and thruster accelerations decreased too, and in the final moments of the engagement, the interceptor attempted to make the miss distance zero by switching the sign of the acceleration.

**Figure 7:**
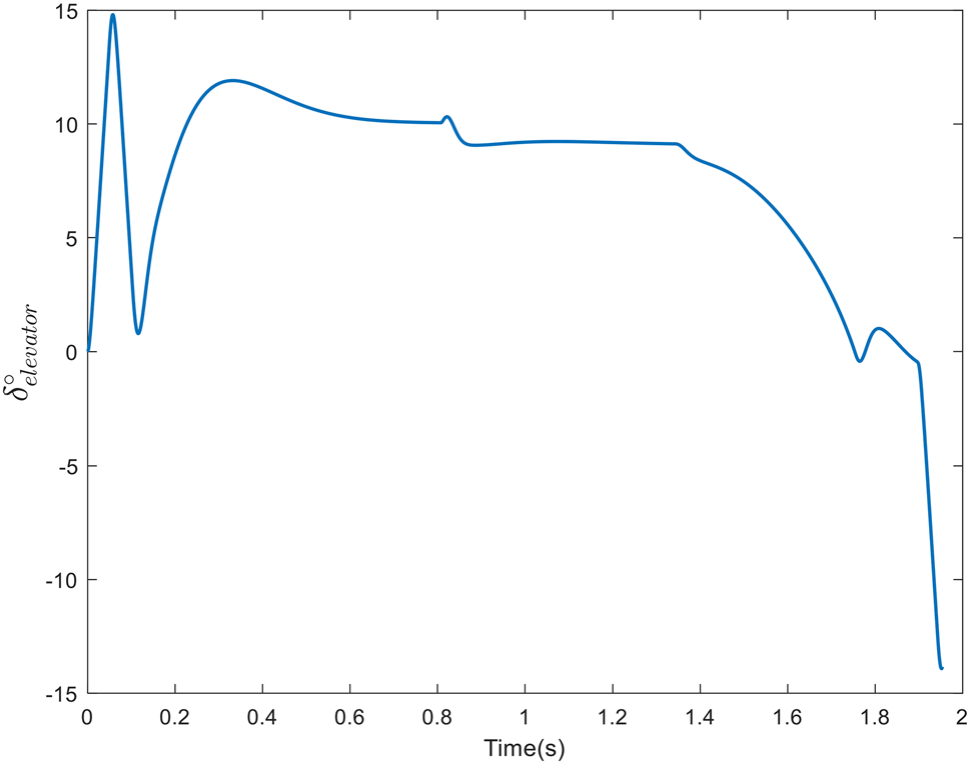
The deflection command in case 1.

**Figure 8:**
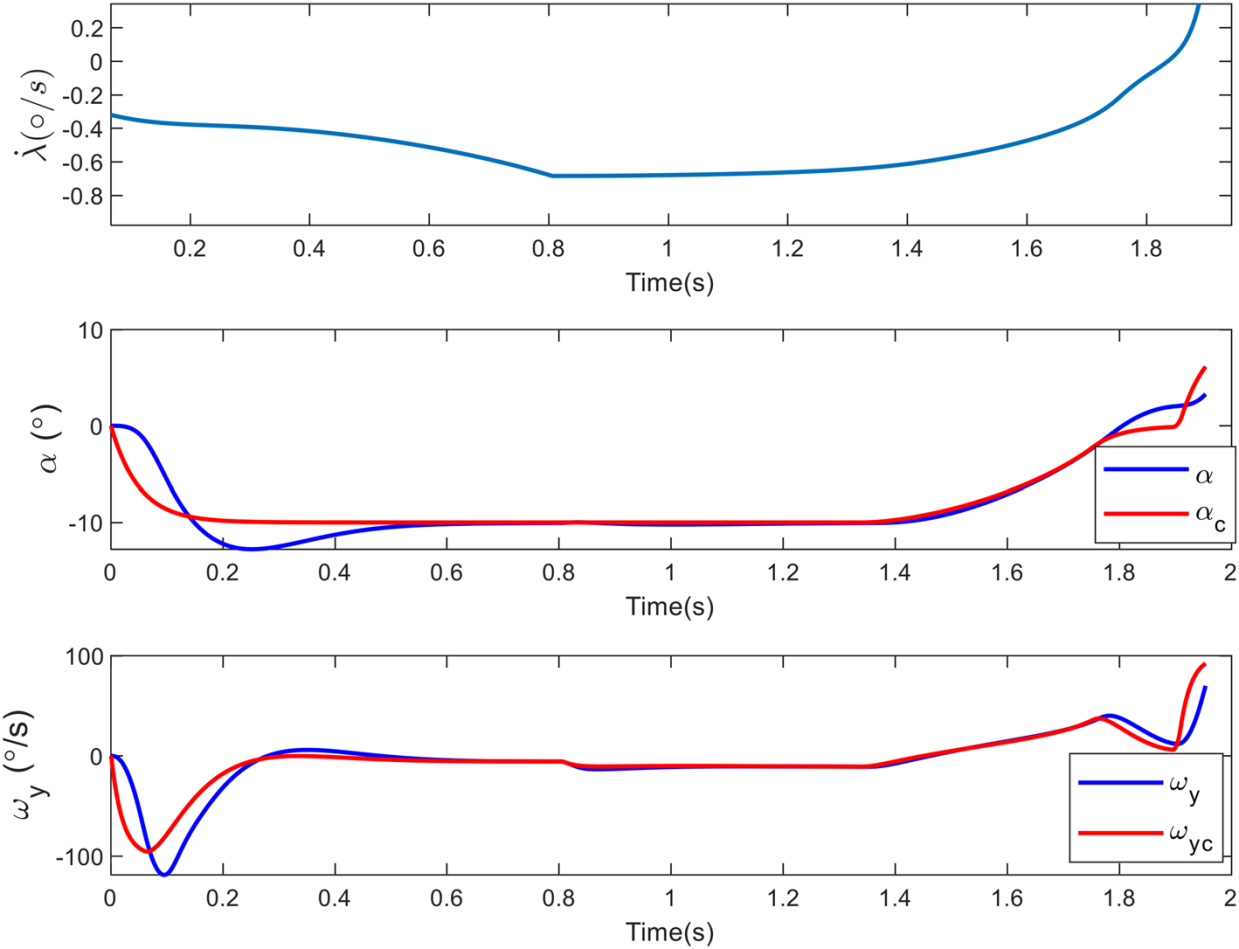
Changes in the angle of attack, pitch rate and LOS rotation rate in case 1.

Figure 7 depicts the elevator steering so that the LOS rotation rate becomes zero and the pitch rate and AOA track the command in the presence of uncertainties, which is illustrated in Figure 8.

In parallel guidance techniques, such as Proportional Navigation (PN), which are based on zeroing the LOS rate, the guidance bandwidth continuously grows by decreasing the relative range^34^. Consequently, the LOS rate changes drastically at the conclusion of the engagement as depicted in Figure 8. Also, this Figure demonstrates that, given a proper time constant, the AOA and angular velocity can follow their commands. Because the inner loop is quicker, the angular velocity tracking error is less than the AOA. Since tracking differentiator is not used, an initial error with command values is created.

**Figure 9:**
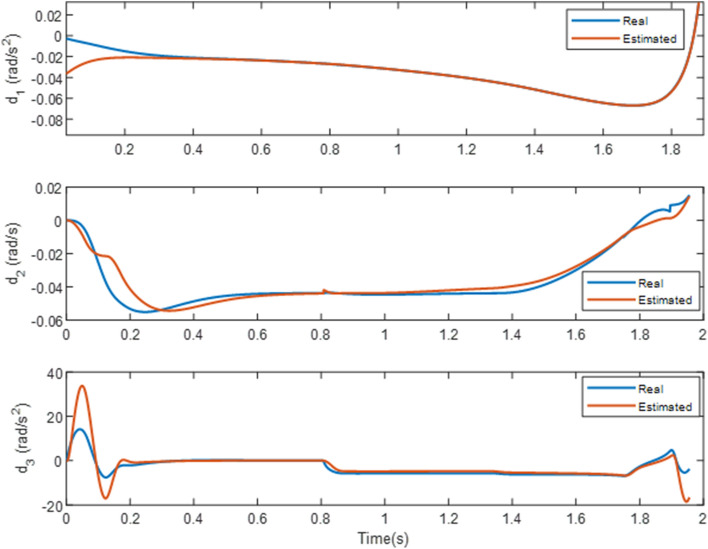
Estimates of disturbances and uncertainties via RESO in case 1.

As it is well-established, using a first-order lag and pseudo-differentiator in the scenario is sufficient and does not cause any problems. Before the thruster was activated, the aerodynamic acceleration could not prevent the increase in the LOS rotation rate. Once the thruster was activated, however, this parameter became zero. Finally, Figure 9 depicts both the estimated and actual values of the disturbance in the problem. As it can be seen, the disturbance leap parameters that occur at 0.85*s* are the activation of the thruster and the torque produced by its distance from the center of mass. The disturbances caused by the target’s drag and acceleration are accurately calculated in $d_1$, and the observer’s bandwidth is suitable for high speeds and short periods.

In this case, it was demonstrated that the proposed method is suitable for use in destroying a ballistic target. Table 3 presents the miss distance for this scenario in four different modes to emphasize the importance of utilizing the thruster and observer in this engagement.

**Table 3:** Comparing the miss distance of different modes in Case 1.

Mode	Scenario	Miss distance (m)
1	Case1	0.46
2	Case 1 without Thruster	15.3
3	Case 1 without RESO	5.13
4	Case 1 without Thruster @ Altitude = 9km	5.54

Accordingly, an observer is required to estimate disturbances and uncertainty for the hit-to-kill interception. The impact of the thruster on the interception at high altitudes is another element of significance included in Table 3. Therefore, the absence of a thruster caused a miss distance of 15.3 m at an altitude of 12 km but only 5.5 m at an altitude of 9 km. This is due to a decrease in target velocity resulting from a decrease in interception altitude, required actuator rate, and interceptor time constant. However, it is not recommended to intercept ballistic targets at low altitudes due to factors such as the target’s increased maneuverability, the increased risk of destruction, and the presence of cluster warheads. The last thing that is investigated in this case is the effect of the controller gains on the miss distance at the height of 12 km. By changing the gains as $k_1=0.5$, $k_2=1$ and $k_3=2$, the simulation results show that the tracking error has increased, but in the presence of the thruster, the miss distance has not changed much and has reached 1.23 m. If the thruster is removed, the miss distance increases to 3.71 m. Also, if the gains are chosen as $k_1=1$, $k_2=5$ and $k_3=25$, due to the increase in the bandwidth of the inner loop compared to the actuator, the simulation becomes unstable and the miss distance becomes 136.2 m.

### Case 2

In this scenario, the key difference is that the LOS rotation rate is passed through the dynamics (44), and the range-dependent error (45) is added and then inserted into the IGC equations. The parameters for the seeker filter are as follows:

51 $$\begin{aligned} \tau =0,\ T=10ms,\ L_1=0.42,\ L_2=12 \end{aligned}$$

**Figure 10:**
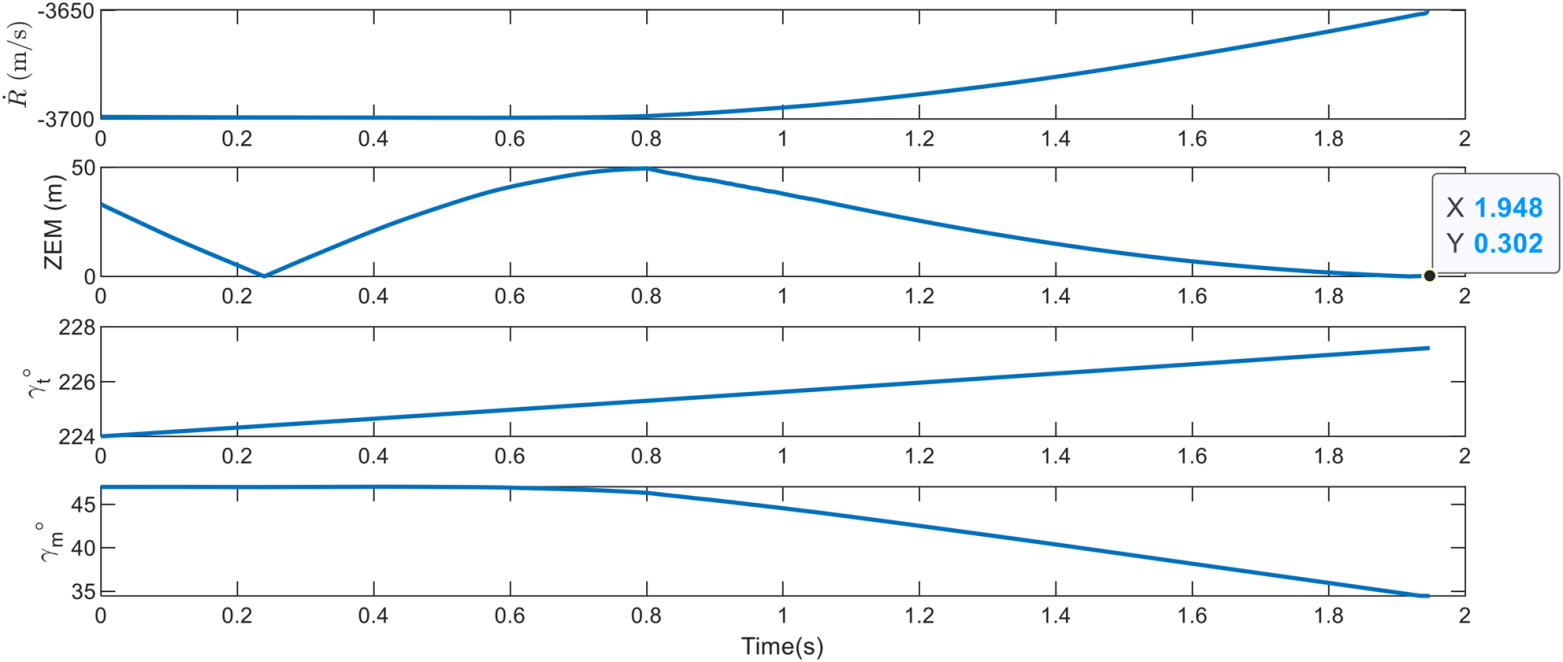
The changes in path angles, ZEM and relative velocity in case 2.

Case 1 interception conditions with $ZEM_{@(t=0) }=33 m$ are used to demonstrate the thruster performance in the presence of seeker error. As ZEM decreases, the miss distance with the ideal seeker and no thruster equals 0.63 m (shifting the target velocity vector by $1^{\circ }$). Currently, the scenario is recreated in two modes, with and without thrusters, and the results are compared using a non-ideal seeker. Figure 10 depicts the variations in the parameters of the target and interceptor throughout the thruster-powered flight.

**Figure 11:**
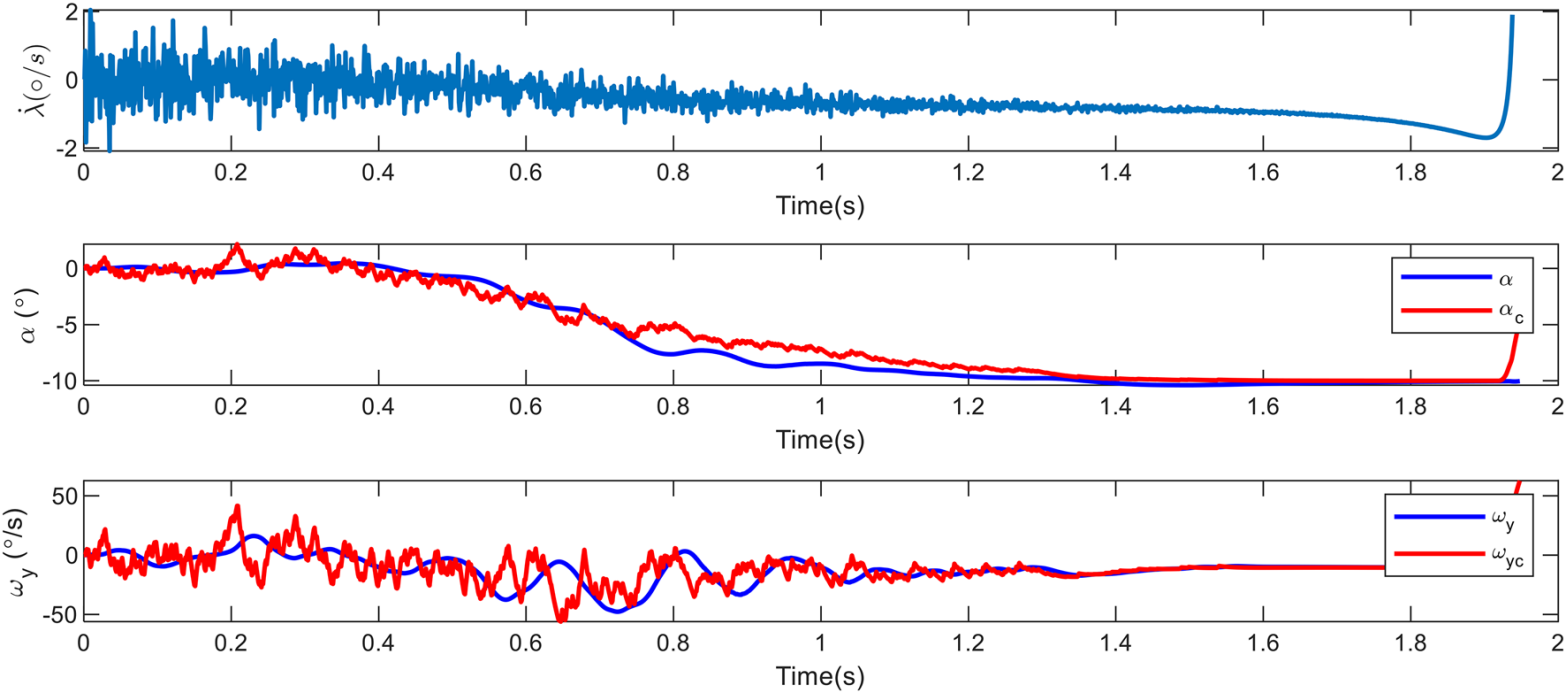
Changes in the angle of attack, pitch rate and LOS rate in case 2.

In this mode, the miss distance corresponds to 0.3 m. Figure 11 illustrates the variations in LOS rotation rate, AOA, and pitch rate.

**Figure 12:**
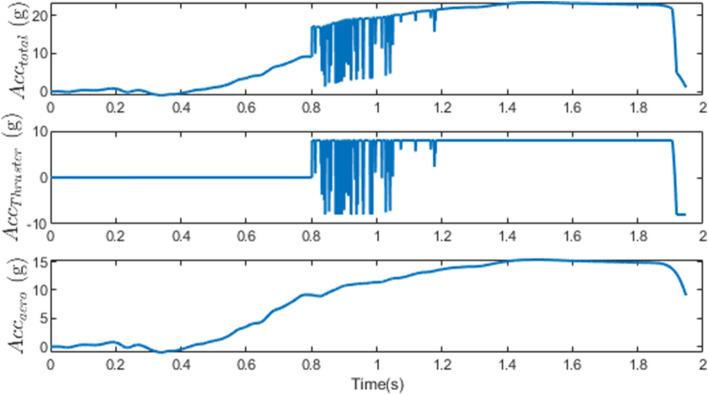
Acceleration of the interceptor due to the angle of attack, thruster activation and the total ones in case 2.

First, due to angle measurement error, the seeker sees the target along the interceptor’s velocity vector, and the interceptor makes no attempt to correct the course. As the distance between the target and interceptor decreases, the seeker’s angle measurement error also decreases. When this accuracy increases, the interceptor has the opportunity to adjust the ZEM. Figure 12 illustrates the changes in aerodynamic and thruster acceleration throughout a flight.

**Figure 13:**
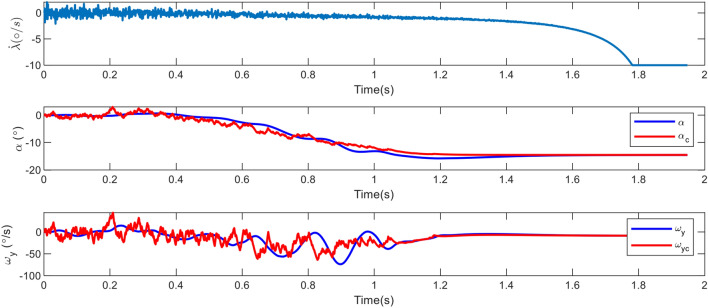
Changes of dynamic variables in Case 2 without thruster.

The thruster pulses may be observed in the incorrect direction due to the seeker’s inaccurate angle measurement during thruster initiation. It has expended all of its energy on ZEM modification by shortening the distance and increasing the precision of the seeker. Replicating the identical scenario without a thruster, results in a miss distance of 22.17 m. In this case, despite the improvement in seeker error and the interceptor’s command to attack at its maximum angle, there was insufficient time to account for the ZEM, and it could not be reduced to zero, as depicted in Figure 13.

**Figure 14:**
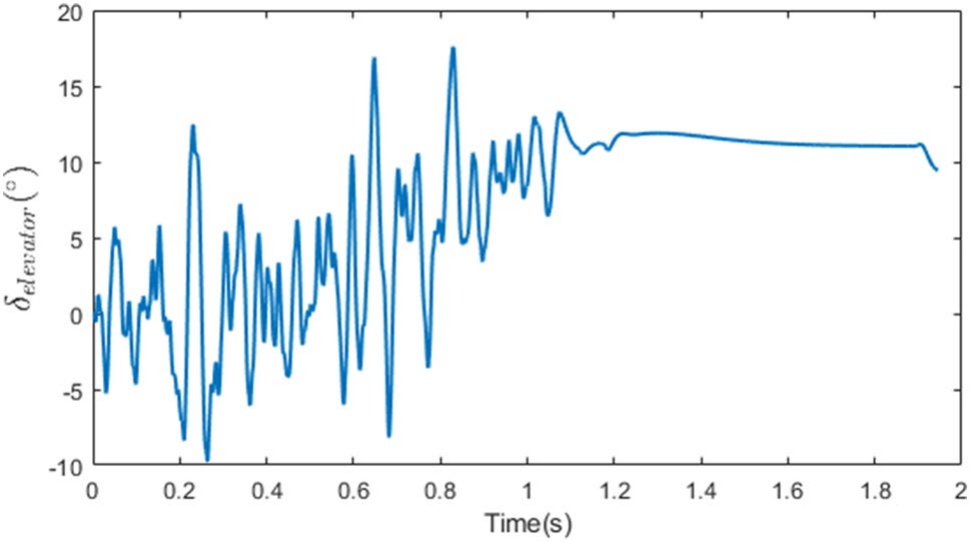
The deflection command in Case 2 without thruster.

In this scenario, the rate of the actuator is particularly important, as its command undergoes rapid dynamics as depicted in Figure 14 due to the noisy behavior of ${\dot{\lambda }}$.

The results indicate that a thruster is required to engage high-speed targets, such as ballistic ones when a non-ideal seeker is present.

### Case 3

**Figure 15:**
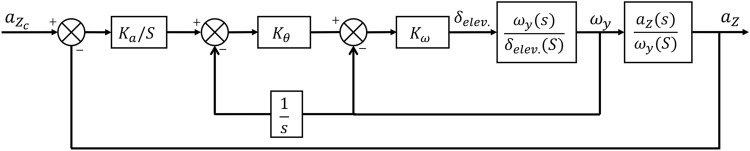
The structure of three-loop autopilot^20^.

In this case, the Monte Carlo execution for various ZEM’s and altitudes is examined to demonstrate the superiority of the proposed approach over the conventional two-loop method. The guidance and control technique described in reference^33^, in which the guidance and control loops are developed independently, serves as a comparison baseline. This method employs the PN law and the three-loop autopilot, as shown in Figure 15.

In this case, the objective was to compare the miss distance between the developed integrated method and the conventional guidance and control one regarding the time lag between the loops. As a result, acceleration was given first to the thruster and then to the three-loop autopilot in the same manner as in the proposed method. In this scenario, a non-ideal seeker was also utilized, as in case 2. No uncertainty was introduced into the problem to reduce the effect of the observer, and the only disturbance was the drag acceleration of the target. Case 3 possessed the same engagement parameters as Case 1.

The three-loop control law is as follows:

52 $$\begin{aligned} \delta _{elevator}=k_{\omega } \omega _y +k_{\omega } k_{\theta }\int \omega _y +k_a k_{\omega } k_{\theta }\int \left( a_Z-a_{z_c}\right) \end{aligned}$$

where $a_Z$ and $a_{z_c}$ are interceptor real and command acceleration in pitch plane, $k_a$, $k_{\omega }$, $k_{\theta }$ denote the control parameters and design as $k_a=2.23$, $k_{\omega }=0.26$, $k_{\theta }=19.3$.

Each point was simulated five times to account for the noise in the seeker measurement, and the mean miss distance at that point was then reported.

**Figure 16:**
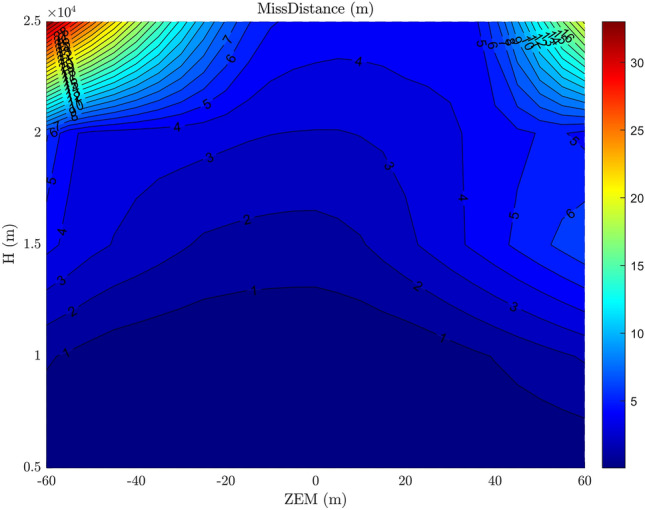
The miss distance contour at different altitudes and ZEMs with integrated guidance and control method.

**Figure 17:**
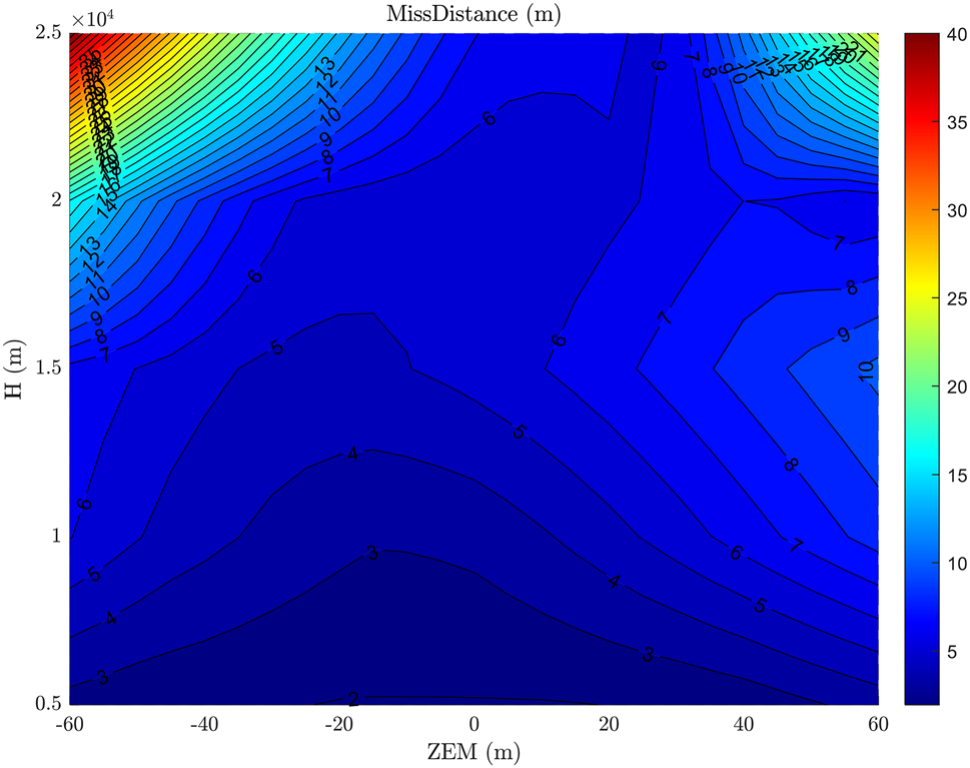
The miss distance contour at different altitudes and ZEMs with the conventional guidance and control method.

As shown in Figs. 16 and 17, the performance of the developed integrated guidance and control method (in identical situations like thruster existence and no uncertainty) is superior to that of the conventional method, despite the time lag existence in both methods. It is due to the consideration of the interceptor’s dynamics in the calculation of AOA command, and required less time lag in the proposed method. This result validates the claim made in subsection 3.1

As can be seen, larger ZEM’s and higher altitudes result in greater increase in miss distance. In addition, the asymmetry of the miss distance around the ZEM is caused by the ballistic target’s drag acceleration.

### **Case 4**

**Figure 18:**
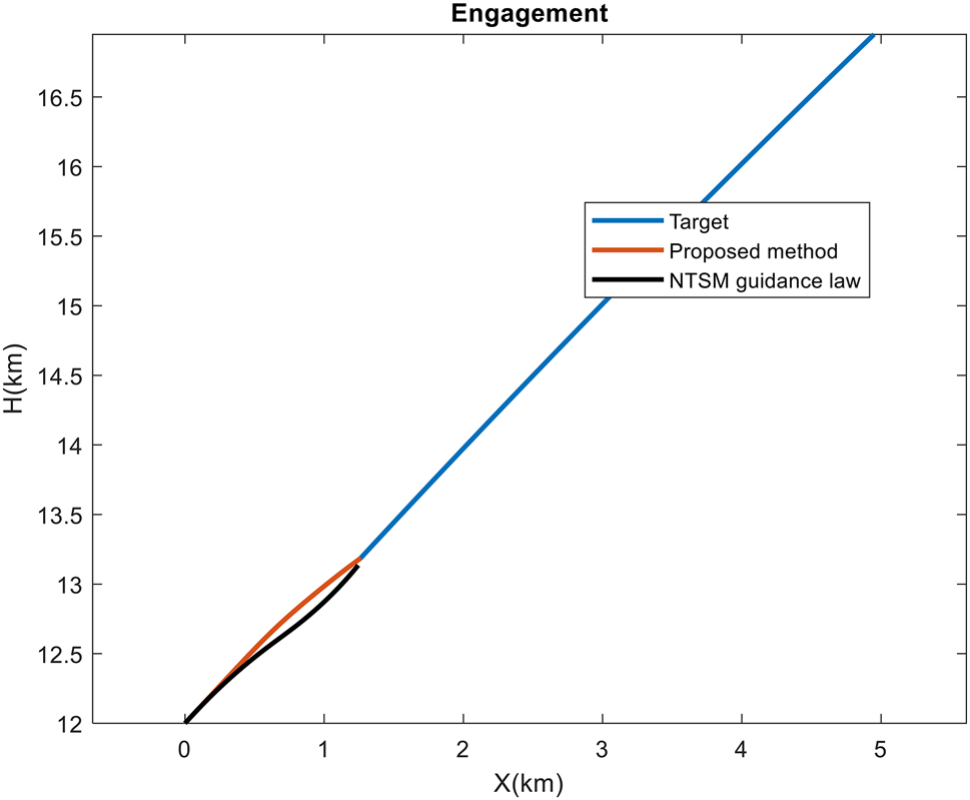
The engagement geometry in Case 4.

**Figure 19:**
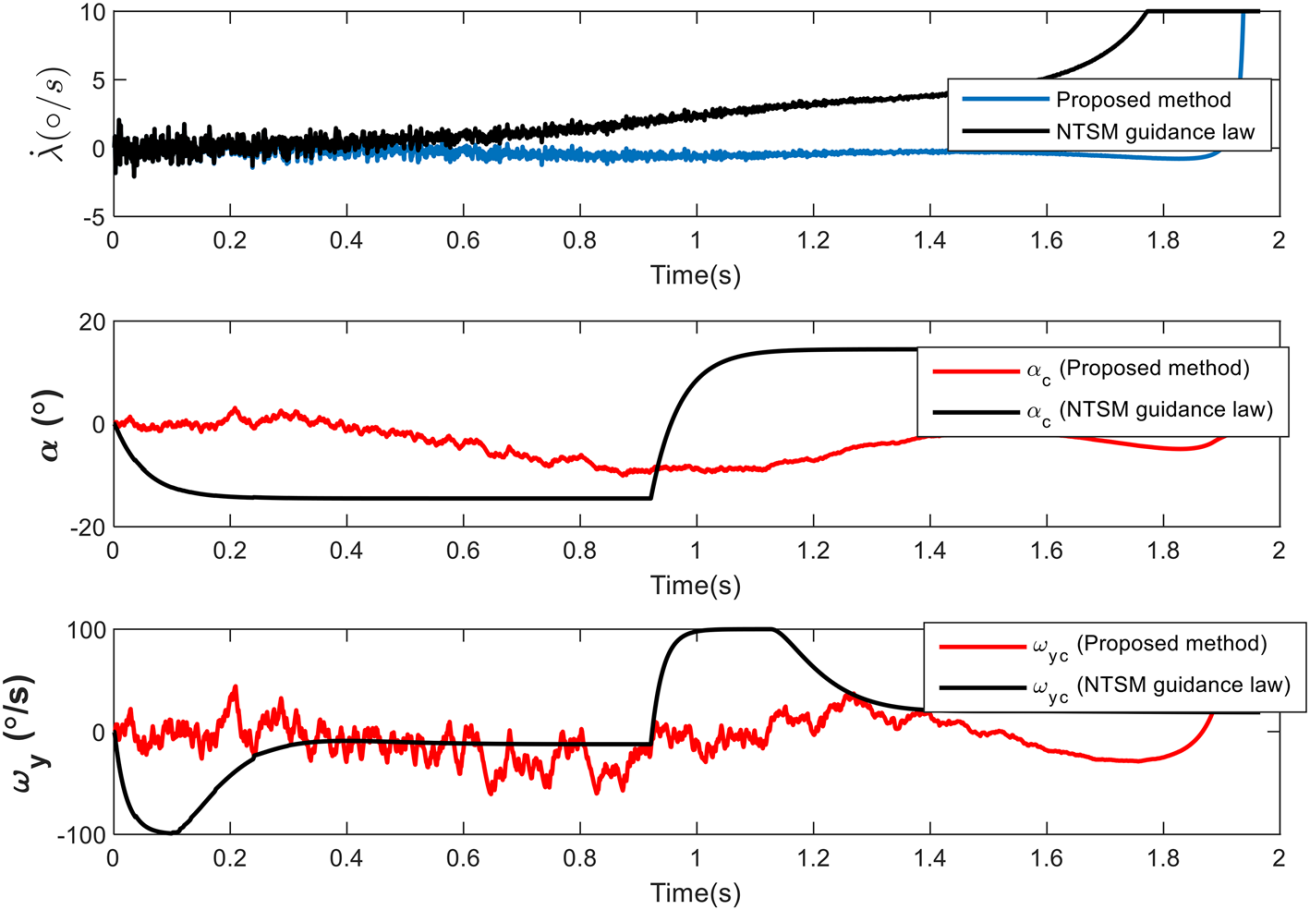
Changes in the angle of attack, pitch rate and LOS rate in Case 4.

In the following explanation, to show the superiority of this method over SMC ones, a comparison with Non-singular Terminal Sliding Mode (NTSM) guidance law, as the main basis of many new research, has been made. To make the comparison fair, this method has been used due to the point that it is developed for the guidance loop against maneuvering targets. We can convert its output acceleration to the $\alpha _c$ and continue the rest of the process as before. Also, the use of thruster, $\hat{d_2}$ and $\hat{d_3}$ are also applied like the proposed method. As a result, the change is only in the way of calculating the $\alpha _c$ and estimating the target maneuver. The NTSM guidance law which is taken from^35^ is presented as follows:

53 $$\begin{aligned} &a_{m}^{c}=\frac{1}{|\cos (\gamma _{m})|} (\frac{R}{\alpha \beta }{\dot{\lambda }}^{2-a}+2|{\dot{R}}|{\dot{\lambda }}) \ + \\&\frac{M}{sign(\cos (\gamma _{m}))}sat((\lambda -\lambda _f)+\beta {\dot{\lambda }}^a) \end{aligned}$$

where $a_{m}^{c}$ is the guidance command, $\lambda _f$ is the desired line-of-sight angle, M=500, $\beta$=10 and a=5/3. The simulation is done for Case 2 scenario (in the presence of seeker noise). Figure 18 illustrates the difference between the paths of proposed IGC and NTSM method. Also, the miss distance of proposed method is 0.3 m (like Case 2) and the miss distance of NTSM method is 18.4 m. Figure 19 depicts the variation in LOS rate, AOA and pitch rate. As mentioned in the introduction section, guidance laws based on SMC that do not have an observer to estimate the target maneuver have problems performing well in a short time. Here too, it is clear that the guidance command is saturated and has caused a drop in performance and an increase in the miss distance.

### Comparison between methods

Using the simulation results, a qualitative comparison can be made between the proposed method, conventional and NTSM ones. In summary, the results of this comparison are shown in Table 4.

**Table 4:** Comparison of characteristics of three simulated methods.

	Proposed IGC	Conventional	NTSM
Easy Implementation	$\times$	$\checkmark$	$\checkmark$
Intercepting maneuvering target	$\checkmark$	$\times$	$\checkmark$
Hit-to-kill ability	$\checkmark$	$\times$	$\times$
Higher bandwidth with the same actuator	$\checkmark$	$\times$	$\times$

## Concluding remarks

This study proposed novel integrated guidance and control method for a dual-controlled interceptor against a tactical ballistic target with back-stepping method based on ADRC structure and daisy-chain procedure for control allocation. The developed method was able to engage with a target at speed of 2500 m/s at an altitude of 13 km in the presence of various disturbances such as drag and high target maneuvering as well as aerodynamic uncertainties with desired miss distance. System stability and asymptotically convergence of tracking error are guaranteed based on the Lyapunov theory.

The simulation results indicated, As the engagement height rises, due to an increase in the interceptor’s time constant and a decrease in the efficiency of the fins, the thruster plays a greater role in the near-miss engagement. In addition, obtaining a low miss distance without a thruster is impossible if a non-ideal seeker, with filter and measurement noise, is used due to the restricted rate of the fin actuator, the high interceptor time constant and the seeker error at the beginning of the engagement.

The contour of the miss distance of various ZEM’s at different altitudes for the conventional method and the proposed one shows that there is a significant improvement in the miss distance in the same conditions.That is the point, because hit-to-kill or near-miss interception against tactical ballistic, is very important for air defense system. Also, the superiority of the proposed method over NTSM guidance law has been shown. In short, the advantage of this method compared to other IGC methods is to simultaneously deal with uncertainties and disturbances without knowing about them, along with achieving proper control performance. This advantage makes it possible to achieve a hit-to-kill scenario in intercepting a ballistic target by using the DCS thruster. Also, the negative point is the use of the Cascade control structure, which causes the bandwidth of each loop to be limited for the proper operation of the next one.

Considering the seeker measurement and actuator delay as an input-output delay in IGC law, using Kalman filter for intercepting a weaving target and integrating seeker filter with IGC law are some ideas for extension of this study in future works.

## Data Availability

All data for reproduction of the manuscript results is available in the ’simulation’ section in text. Any additional data is available upon request. For this purpose, contact alichitsaz@aut.ac.ir
